# Evolutionary escalation in an exceptionally preserved Cambrian biota from the Grand Canyon (Arizona, USA)

**DOI:** 10.1126/sciadv.adv6383

**Published:** 2025-07-23

**Authors:** Giovanni Mussini, James W. Hagadorn, Anne E. Miller, Karl E. Karlstrom, Rhydian Evans, Carol M. Dehler, Salvador Bastien, Nicholas J. Butterfield

**Affiliations:** ^1^ University of Cambridge, Department of Earth Sciences, Downing St., Cambridge CB2 3EQ, UK.; ^2^ Denver Museum of Nature and Science, 2001 Colorado Blvd., Denver, CO 80205, USA.; ^3^ Science and Resource Management, Grand Canyon National Park, 1824 S Thompson St. STE 200, Flagstaff, AZ 86001, USA.; ^4^ Department of Earth and Planetary Sciences, University of New Mexico, 221 Yale Blvd NE, Albuquerque, NM 87131, USA.; ^5^ Independent Researcher, London, UK.; ^6^ Department of Geology, Utah State University, Logan, UT 84322, USA.

## Abstract

Exceptionally preserved fossil assemblages, or Konservat-Lagerstätten, open direct windows on non-biomineralized faunas that chronicle the Cambrian radiation of animal phyla. However, these assemblages do not typically capture the well-oxygenated, resource-rich environments sustaining most metazoan diversity in modern marine systems. We describe exceptionally preserved and articulated carbonaceous mesofossils from the middle Cambrian (~507 to 502 million years) Bright Angel Formation of the Grand Canyon (Arizona, USA). This biota preserves probable algal and cyanobacterial photosynthesizers together with a range of functionally sophisticated metazoan consumers: suspension-feeding crustaceans, substrate-scraping molluscs, and morphologically exotic priapulids with complex filament-bearing teeth, convergent on modern microphagous forms. The Grand Canyon’s extensive ichnofossil and sedimentological records show that these phylogenetically and functionally derived taxa occupied highly habitable shallow-marine environments, sustaining higher levels of benthic activity than broadly coeval macrofossil Konservat-Lagerstätten. These data suggest that evolutionary escalation in resource-rich Cambrian shelf settings was an important driver of the assembly of later Phanerozoic ecologies.

## Teaser

New Cambrian fossil biota from the Grand Canyon captures a “Goldilocks zone” of early animal life.

## INTRODUCTION

The essential elements of Phanerozoic marine ecosystems became established in the Cambrian period (*1*). Burgess Shale–type (BST) macrofossil deposits, epitomized by the middle Cambrian [~505 million years (Ma) old] Burgess Shale of British Columbia, open a direct window on the non-biomineralized organisms that dominated Cambrian communities, typically preserving them as carbonaceous compressions (*2*–*5*). “BST biotas” or “BST faunas” denote both this distinctive taphonomic window and a loose set of taxonomically overlapping assemblages dominated by stem-group relatives of living phyla (*2*, *5*). These biotas contain few examples of the modern taxonomic classes and derived lifestyles (e.g., substrate miners, zooplanktonic suspension feeders, and a multitiered epifauna) that typify later Paleozoic communities (*6*) despite emerging evidence for protracted Cambrian origins of some of these habits [e.g., (*7*–*9*)].

However, exceptional preservation of BST-macrofossil biotas typically corresponds to similarly “exceptional” environmental conditions, with limited degradation and reworking of carcasses by bioturbators, scavengers, and decomposers (*3*, *4*). Most early Cambrian BST-macrofossil biotas, which sample slope to prodelta environments (e.g., the Chengjiang, Emu Bay Shale, and Sirius Passet Lagerstätten), show evidence for dysoxic to anoxic burial conditions (*10*–*13*). Middle Cambrian counterparts record similarly oxygen-limited offshore settings: Among them are the outer detrital deposits of the Burgess Shale (*4*, *5*); the Wheeler, Marjum, and Weeks biotas of Utah (*14*); and the Kaili biota of China (*4*, *15*–*17*). The Spence Shale of Utah and Idaho records a broader inner shelf to offshore gradient (*18*); nonetheless, its bottom waters were at least intermittently dysaerobic (*19*–*21*). Organically preserved, submillimeter-scale small carbonaceous fossils (SCFs) (*3*) have further extended the range of BST metazoans into shallow epicratonic (*3*) to episodically subaerial (*22*) settings. However, studies on these (typically disarticulated) body fossils often lack detailed interpretation of associated trace fossils and environmental indicators (*3*, *23*–*26*)—or sample habitats colonized only by the most ecophysiologically tolerant metazoans (*22*). The result of this combination of taphonomic biases and unequal study efforts across paleoenvironments is an overrepresentation of ecologically “marginal” settings by Cambrian biotas preserving non-biomineralized taxa.

This biased paleoenvironmental coverage affects our ability to test macroevolutionary hypotheses about the assembly of Phanerozoic ecologies. In particular, in stable, resource-rich environments hosting structured trophic networks, the main agents of selection tend to be other organisms rather than extrinsic abiotic factors (*27*, *28*). Therefore, such settings may be more conducive to evolutionary escalation (*28*–*31*): the directional, open-ended ratcheting of adaptive innovations against biotic “enemies” (*28*–*32*), ranging from trophic competitors to predators and pathogens. Its result is a long-term elevation of biological “performance standards” (*28*) in resource acquisition and maintenance, driving the assembly of increasingly elaborate means of defense, offense, and food or energy capture. In turn, these innovations can unlock and inject a wider share of resources into the broader ecosystem, promoting further escalation (*28*, *29*, *33*). In its self-amplifying, open-ended nature, escalation differs from both “court jester” scenarios, which posit extrinsic abiotic forcings as first-order drivers of macroevolution, and alternative zero-sum coevolutionary models postulating a fixed share of available resources, such as the Red Queen hypothesis (*29*). Unlike zero-sum models, the escalation hypothesis predicts that the elevation of biotic performance standards does not generally lead to the simultaneous extinction of less competitive taxa; instead, it relegates them in step-wise fashion to progressively more resource-poor, physiologically marginal settings as ecological baselines are ratcheted up by derived competitors (*28*, *30*, *31*).

Constructing and maintaining complex adaptations for resource acquisition and retention requires outlays of energy; therefore, escalation is considered more likely to be initiated under evolutionarily permissive (*28*) conditions, whereby intense biotic competition takes place in arenas of plentiful and accessible nutrients, oxygen, and energy sources. These settings can sustain open-ended antagonistic coevolution and provide sufficient resources to bypass physiological trade-offs, promoting the buildup of increasingly elaborate, high-investment adaptive traits (*30*). However, the macroevolutionary impact of escalation remains incompletely understood, not least because of the relative paucity of non-biomineralized faunas [compare with (*32*)] and discontinuous paleoenvironmental coverage (*29*) of the fossil record. To date, Cambrian escalation has been tested almost exclusively through the lens of the shelly fossil record rather than the more patchily preserved, but more broadly representative record of non-biomineralizing organisms (*32*, *34*). If escalation had a pervasive influence on the trajectory of early Phanerozoic evolution, Cambrian non-mineralized faunas under more permissive conditions may be predicted to show both adaptations for higher performance standards of resource acquisition (*28*) and greater taxonomic and functional overlap with later Paleozoic successors, compared to biotas from coeval marginal habitats (*4*, *22*).

**Fig. 1.**
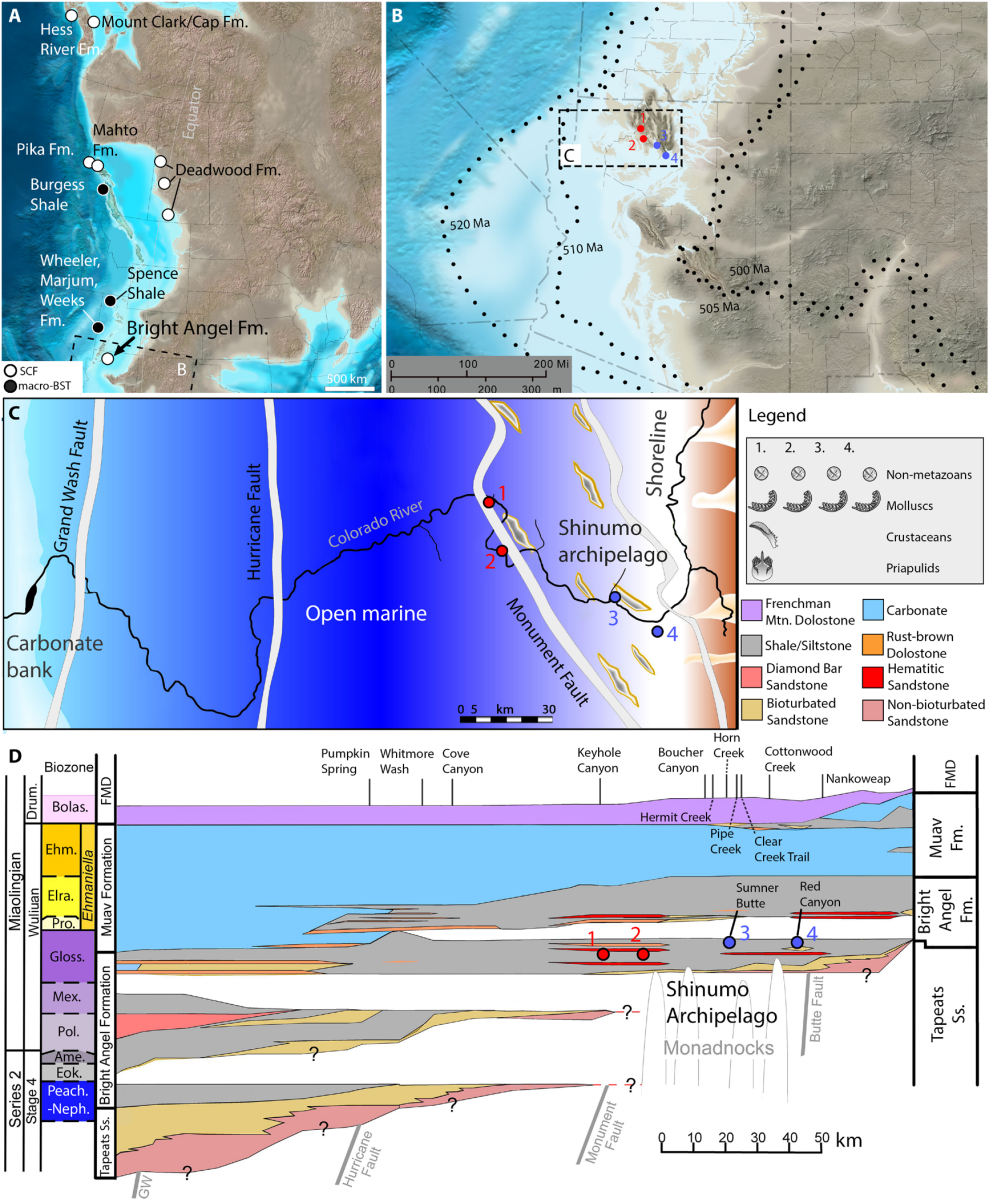
Paleogeography, paleoenvironment, and lithology of the BAF. (**A**) Reconstruction showing paleoenvironmental map of western North America in the Middle to Late Cambrian (~505 to 495 Ma), showing locations of major exceptionally preserved biotas (*14*, *18*, *22*–*25*, *51*, *60*); Deadwood Formation localities, coming from later time intervals, are not from terrestrial settings. Source map 2023 Colorado Plateau Geosystems Inc. (**B**) Detail of boxed area in (A), broadly corresponding to present-day Arizona. Dotted lines denote transgressive progression of shorelines (520 to 500 Ma), from (*40*); colored dots correspond to fossil locations 1 to 4 as shown in (C). (**C**) Paleoenvironmental reconstruction of the Grand Canyon area at the time of deposition of the Bright Angel biota (~505 Ma), showing positions of the present-day Colorado river and major faults for orientation. Red dots indicate locations of the SCF biota described herein; blue previously described assemblages from the Red Canyon and Sumner Butte localities recovered through standard palynological processing (*37*). Legend on the right summarizes major organically preserved fossils recorded at each locality. (**D**) Wheeler diagram of the Tonto Group after (*36*), showing Cambrian Series, stages, trilobite biozones, and main lithologies as indicated by legend on the right [which is specific to (D)]. Localities shown in fig. S11, situated near studied ichnofossil sections, are marked by name on the horizontal axis. Vertical axis is based on relative thickness of zones; question marks indicate that basal clastics lack biostratigraphic and geochronologic control beyond maximum depositional ages (*36*). Disconformities identified by missing trilobite bizones. FMD, Frenchman Mountain Dolostone; GW, Grand Wash fault; Ss, sandstone; Peach.-Neph., *Peachella iddingsi* to *Nephrolenellus multinodus*; Eok., *Eokochaspis nodosa*; Ame., *Amecephalus arrojosensis*; Pol., *Poliella denticulata*; Mex., *Mexicella mexicana*; Gloss., *Glossopleura walcotti*; Pro., *Proehmaniella*; Elra., *Elrathiella*; Ehm., *Ehmaniella*; Bolas., *Bolaspidella*; Ced., *Cedaria*; Crep., *Crepicephalus*.

Here, we describe a middle Cambrian biota under comparatively “permissive” conditions: an oxygenated, fully marine shelf setting marked by abundant traces of metazoan activity relative to broadly coeval macrofossil Lagerstätten. We report exceptionally preserved priapulid, crustacean, and molluscan SCFs from the lower to middle Bright Angel Formation (BAF) (~505 Ma) (*35*, *36*) in Grand Canyon National Park (Arizona, USA), representing a nearshore-offshore bathymetric gradient (Fig. 1, A to C) hosting well-aerated, extensively bioturbated normal marine shelf environments (*36*–*39*) . Microstructural data from the Bright Angel SCFs complement the Grand Canyon’s biomineralized and trace fossil records to reveal an unusually wide range of coexisting derived taxa and sophisticated feeding adaptations, in a deposit no more than ~3 Ma younger than the Burgess Shale.

## RESULTS

### Geological context

The Grand Canyon’s oldest Phanerozoic strata are recorded by a five-member, unmetamorphosed Cambrian sedimentary package (Fig. 1D) known as the Tonto Group (*36*, *40*). The latter was deposited on the subequatorial margin of the North American craton during the early Paleozoic Sauk 2 marine transgression (Fig. 1, A and B) (*40*, *41*). The upper series 2 to Miaolingian [*~*507 to 502 Ma (*40*)] BAF (Fig. 1, C and D) lies in the lower Tonto Group (*40*). It is conformably underlain by the ≤508.19 ± 0.39 Ma to ≤506.64 ± 0.32 Ma Tapeats Sandstone (*40*), a succession of cliff-forming, mostly cross-bedded tan to red-brown sandstone (*36*), and punctuated by two disconformities before grading into the limestone-dominated, *~*502- to 499-Ma Muav Formation (*36*, *40*, *42*).

In the Grand Canyon, the BAF is a *~*100-m-thick formation consisting predominantly of greenish glauconitic to red shale with interbedded siltstone, sandstone, and lesser dolostone (Fig. 1D). The BAF’s depositional environment can be broadly characterized as nearshore-offshore transitional (*36*, *38*, *42*). In the eastern Grand Canyon (cratonward; Fig. 1, A to C), the BAF’s cyclically thickening and thinning heterolithic sandstone beds suggest storm- and tide-influenced settings, consistent with their cross-bedded, ripple-laminated, and wavy to lenticular morphologies (*37*, *38*, *42*, *43*). Westward (oceanward), increasingly frequent carbonates and iron-rich glauconitic shale beds point to a gently deepening shelf (*36*, *38*, *39*). Extensive interfingering of “marginal/nonmarine” to fully marine deposits along this east-west gradient (Fig. 1, B and C) indicates a mosaic of emergent islands, shoals, embayments, and tidal channels that were covered by the Sauk 2 transgression (*36*, *37*, *40*, *41*).

In the Grand Canyon, the BAF hosts a rich and relatively well-characterized ichnofossil record comprising at least 21 ichnogenera [see (*39*, *44*)]. Traces are abundant in both shale and sandstone/siltstone-dominated horizons (*37*, *39*, *44*) and distributed across two intergrading ichnofacies: the *Skolithos* and *Cruziana* ichnofacies (*37*, *39*, *44*). Vertical suspension feeding burrows dominate the *Skolithos* ichnofacies, typical of foreshore to middle shoreface deposits (*37*, *39*, *45*). The *Cruziana* ichnofacies, characterizing fully marine, lower shoreface to lower offshore deposits (*37*, *39*, *44*, *45*), hosts more disparate vermiform and arthropod-type feeding, locomotory, and resting traces (Supplementary text).

In contrast to this diverse ichnological record, body fossils in the BAF have been limited to biomineralizing taxa (fig. S1), including trilobites, brachiopods, hyoliths, eocrinoids, bradoriids, and chancelloriids (*37*, *42*, *46*, *47*). These fossils are sporadically distributed but locally abundant in individual quarries (*46*). Metazoan “worms” have not been found in the BAF as body fossils, though vermiform traces comprise 77% of its recorded ichnotaxa (Supplementary text) (*39*). Conventional palynological sampling of the eastern BAF (Fig. 1C) has also yielded metazoan (e.g., radula) fragments, spheroidal acritarchs, organic filaments, and hypothesized cryptospores (Supplementary Text and fig. S2) (*3*, *37*).

### Small carbonaceous fossils

**Fig. 2.**
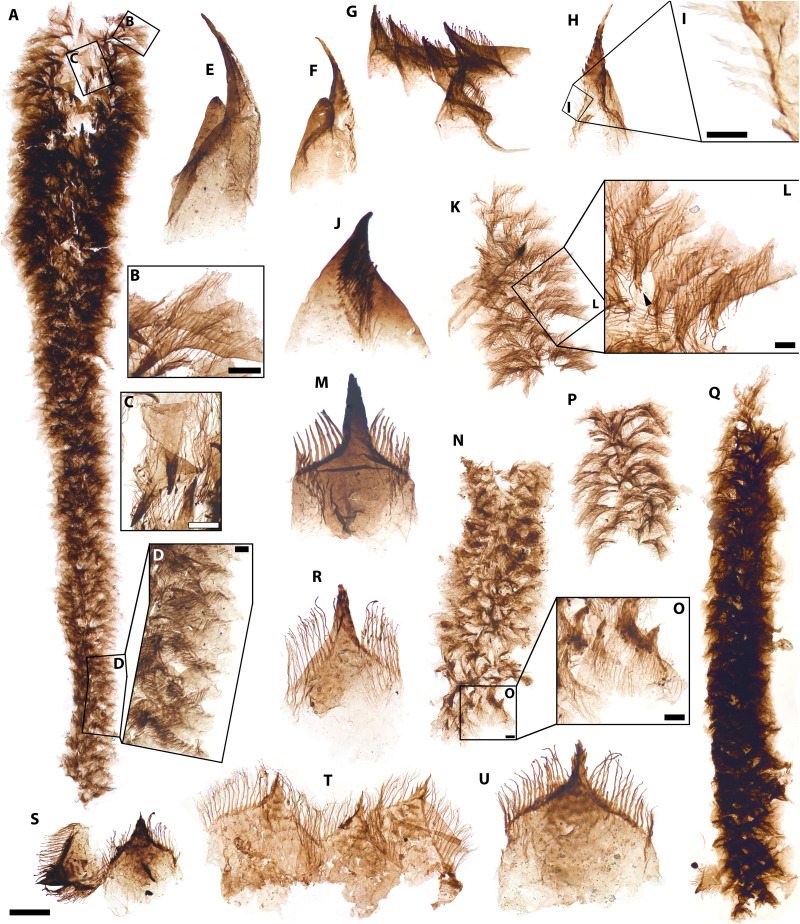
*Kraytdraco spectatus* gen. et sp. nov., proximal (i.e., narrow) pharyngeal tract. (**A**) Articulated proximal tract showing flaring proximal region and possible tapering distal “funnel.” (**B**) Detail of boxed area in (A), showing teeth of the flaring region in side view. (**C**) Detail of boxed area in (A), showing teeth of the flaring region in frontal view and denticles with club-like terminations. (**D**) Detail of boxed area in (A), showing teeth of the tapering funnel with complex filamentous denticles. (**E** and **F**) Introvert hooks with associated cone-like scalids. (**G**) Proximal teeth from the flaring region showing falcate profile and club-like terminations of denticles. (**H**) Isolated introvert hook. (**I**) Detail of boxed area in (H), showing filamentous projections of the basal denticles. (**J**) Tooth morphotype showing falcate shape. (**K** and **P**) Semi-articulated filamentous teeth from the narrow tract. (**L**) Detail of boxed area in (K) showing teeth in side view, with club-like to laterally branching denticles but already showing a multicuspidate apex (black arrowhead). (**M**) Tooth intermediate between those of the “flaring” and funnel-like regions, showing bifurcating basal denticles and apical denticles with lateral tuberculate projections. (**N**) Articulated filamentous teeth from the narrow tract. (**O**) Detail of boxed area in (N), showing relatively distal teeth with fine side-branches and multicuspidate apex. (P) Semi-articulated proximal teeth. (**Q**) Articulated funnel-like region of the narrow tract. (**R**) Tooth from the funnel-like region showing finely branching apical and basal denticles. (**S** to **U**) Distal-most teeth of the narrow tract, showing finely branching denticles, broadly equilateral profiles, transverse comb-like striations and multicuspidate prongs. Slide numbers and England Finder coordinates listed in data S1. Scale bars, (A and Q) 100 μm; (B, C, J, M, and R) 20 μm; (D, I, L, and O) 10 μm; (E, F, H, K, N, P, and S) 60 μm; (G) 50 μm; (T) 25 μm; (U) 45 μm.

SCFs are non-biomineralized, organic-walled micro- to mesofossils with a recalcitrant extracellular matrix (*3*). Their emerging Cambrian record is increasingly complementing the paleoecological and biogeographical data from conventional palynomorphs, BST macrofossils, mineralized body fossils, and ichnofossils (*3*, *48*). In the BAF, SCFs show minimal metamorphic alteration, reflected by light colorations consistent with low thermal maturity and submicrometer-scale preservation of delicate morphological structures (e.g., Fig. 2).

We recovered a total of 1539 SCFs from the BAF (data S1). These specimens came from rock samples corresponding to the low-energy, upper offshore/lower shoreface depositional environments of the lower to middle BAF (*36*, *38*). Studied localities lie in the central Grand Canyon region, bordering the bedrock islands of the Shinumo Archipelago (Fig. 1, C and D) and record typical BAF facies of unbioturbated glauconitic shales interbedded with bioturbated layers at centimeter to decimeter-scale intervals. We extracted SCFs from two fist-sized, in situ samples of moderately micaceous, light green-gray and finely laminated glauconitic shales, collected at the locations and approximate stratigraphic heights shown in Fig. 1 (C and D). Each sample spanned no more than ~15 cm of vertical thickness. Of the recovered SCFs, 967 are attributed to priapulids, a phylum of radially symmetrical ecdysozoan worms with a tripartite body subdivided into a posterior trunk, a retractile introvert, and an eversible toothed pharynx (*49*). Another 11 recovered metazoan SCFs are attributed to molluscs and 201 to arthropods. We also recovered 95 sphaeromorphic acritarchs and 7 aggregates of filamentous microfossils (fig. S3 and data S1). We extracted most of these SCFs (*N* = 1527), including all unambiguous arthropod and priapulid specimens, from a hyperproductive shale sample from the most westward (i.e., oceanward) fossiliferous locality (locality 1 in Fig. 1, C and D); the remainder of the recovered SCFs, including molluscan and non-metazoan or unidentified specimens, are from a sample coinciding with locality 2, as shown in Fig. 1 (C and D) (data S1).

**Fig. 3.**
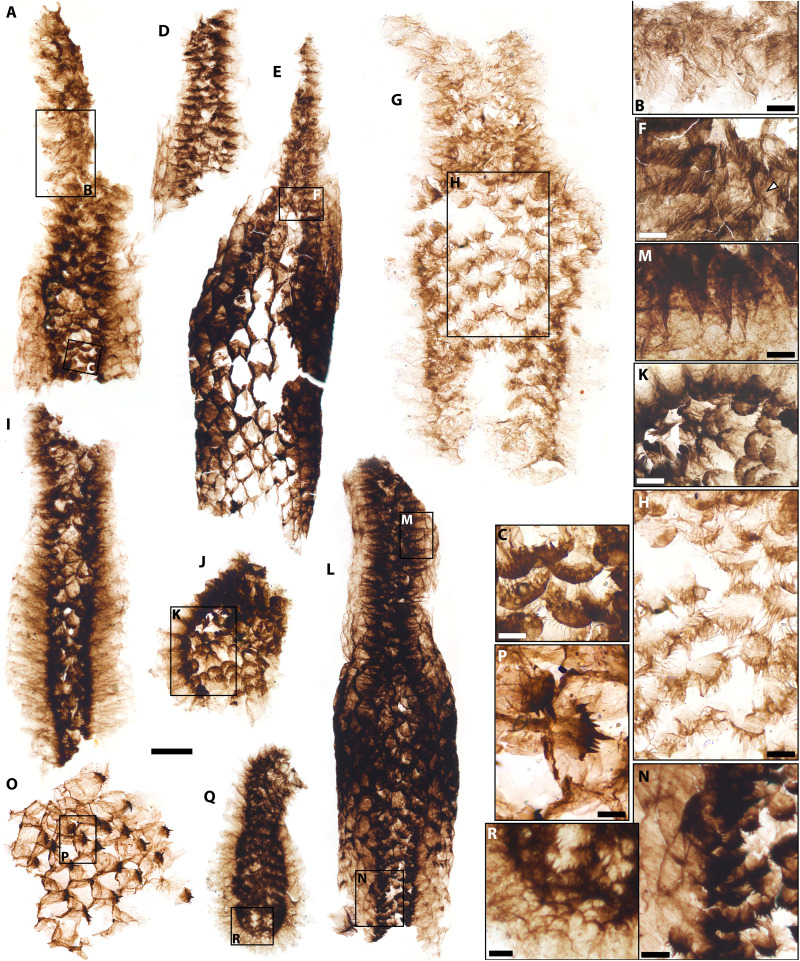
*Kraytdraco spectatus* gen. et sp. nov., distal (i.e., wide) pharyngeal tract and its connection to the narrow tract. (**A**) Connection between narrow (top) and wide (bottom) tracts. (**B**) Detail of boxed area in (A), showing filament-bearing teeth in the narrow tract. (**C**) Detail of boxed area in (A), showing U-shaped filament-bearing teeth transitional between the morphologies characterizing the narrow and wide tracts. (**D**) Portion of narrow tract bearing polygonally patterned external cuticle. (**E**) Articulated section of the narrow tract (top) connected to an expanding zone (proximal-most region of the wide tract) covered by polygonally patterned cuticle. (**F**) Detail of boxed area in (E) showing densely filamentous teeth of distalmost narrow tract. (**G**) Small partly articulated specimen (a possible juvenile) showing partly articulated narrow (top) and wide (bottom) tracts. (**H**) Detail of boxed area in (G) showing transition between U-shaped filament-bearing teeth [top, cf. (C)] and more distal teeth bearing conical spine-like denticles (bottom). (**I** and **J**) Proximal sections of the wide tract. (**K**) Detail of boxed area in (J) showing filament-bearing teeth with transverse comb–like striations, adjacent to U-shaped teeth with conical denticles. (**L**) Specimen comprising sections of narrow and wide tracts, surrounded by polygonally patterned cuticle. (**M**) Detail of boxed area in (L) showing filament-bearing teeth with transverse comb–like striations. (**N**) Detail of boxed area in (L) showing distal teeth with spine-like denticles. (**O**) Teeth with spine-like denticles and associated polygonally patterned cuticle. (**P**) Detail of tooth in (O), showing conspicuous medial prong. (**Q**) Distalmost termination of the pharynx. (**R**) Detail of teeth bearing spine-like denticles in (Q). Slide numbers and England Finder coordinates listed in data S1. Scale bars, (A, D, E, I, J, L, and Q) 100 μm; (B, F, M, and K) 10 μm; (C, H, and P) 5 μm; (G, R, and N) 20 μm; (O) 30 μm.

**Fig. 4.**
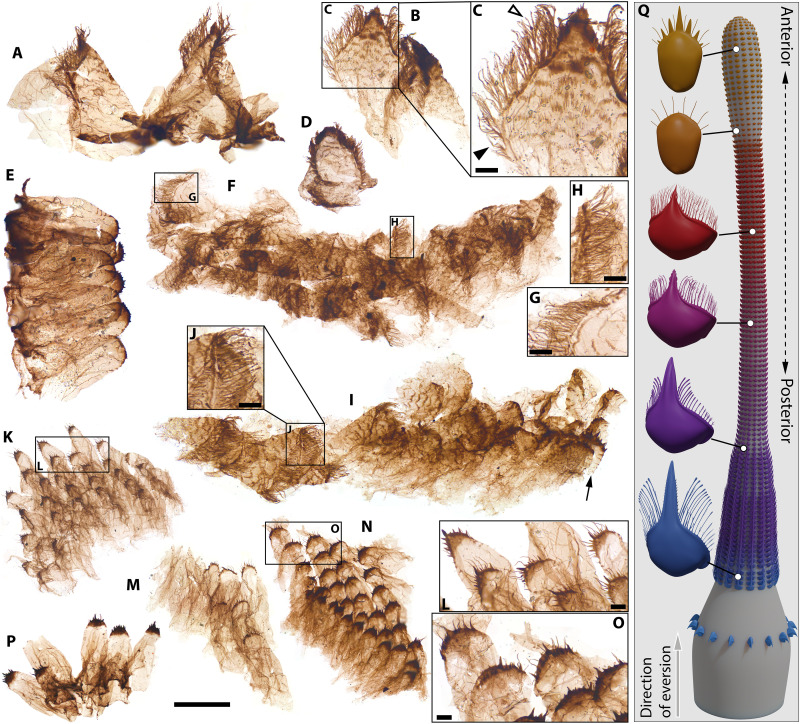
*Kraytdraco spectatus* gen. et sp. nov., teeth in the transitional region between the narrow and wide pharyngeal tracts, and reconstruction of the proboscis. (**A** and **B**) Subtriangular transitional teeth with elaborate filamentous denticles and multicuspidate prong. (**C**) Detail of boxed area in (B) showing filamentous denticles, multicuspidate prong, and transverse comb–like projections; basally splintered denticles are indicated by black arrowhead, apical denticles with side branches by white arrowhead. (**D**) U-shaped (more distal) transitional tooth. (**E**) Distal U-shaped transitional tooth, showing incipient spine-like denticles and transverse comb–like striations laterally. (**F**) Articulated section of subtriangular to U-shaped transitional teeth, with insets showing details of basally splintered (**G**) and bifurcating/laterally branching denticles (**H**). (**I**) Articulated section of transitional teeth bearing filamentous (left) to spine-like (right, arrowed) denticles. (**J**) Detail of boxed area in (I), showing filamentous denticles. (**K** to **P**) Clusters of distal teeth combining spine-like denticles with interspersed unbranched filaments; details of apical regions shown in (L) and (O). (**Q**) Idealized reconstruction of the everted pharynx and anteriormost introvert of *Kraytdraco spectatus* gen. et sp. nov, based on the SCFs shown in Figs. 2 to 4. Schematic models of representative tooth types (left) are color coded on the basis of their inferred anteroposterior placement on the everted pharynx, as shown by the associated connecting lines. Artist Credit: Rhydian Evans. Slide numbers and England Finder coordinates listed in data S1. Scale bars, (A, B, D, and I) 50 μm; (C, G, H, and J) 5 μm; (E and F) 60 μm; (K, M, N, and P) 100 μm; (L and O) 10 μm.

Priapulid pharyngeal teeth (Figs. 2 to 4 and figs. S4 to S8) are U- to V-shaped sclerites projected forward when the pharynx is everted to capture food (*49*, *50*). These elements show a diagnostic combination of features: a ridge-like marginal “arch,” a basal “pad” of thinner cuticle, a spinose, distally directed terminal “prong,” and lateral “denticles” consisting of hair- or spine-like projections extending from the arch and pointing distally (*49*, *51*, *52*). Honeycomb-like sheaths surrounding the pharyngeal teeth (Fig. 3, D, L, and O, and fig. S4, H, N, and O) are directly comparable to the polygonally patterned cuticles of extant and Cambrian priapulids (*53*, *54*). Sclerites bearing an arch with longitudinal rows of denticles, but having a characteristically falciform profile with a markedly concave margin (e.g., Fig. 2, E, F, and H), record posteriorly pointing “hooks” from the introvert or trunk regions of priapulids, comparing well with extant and macrofossil specimens that have a similar aspect ratio, degree of curvature, and denticulate ornament (*49*, *51*, *52*); see section “Small carbonaceous fossils, Priapulids.”

**Fig. 5.**
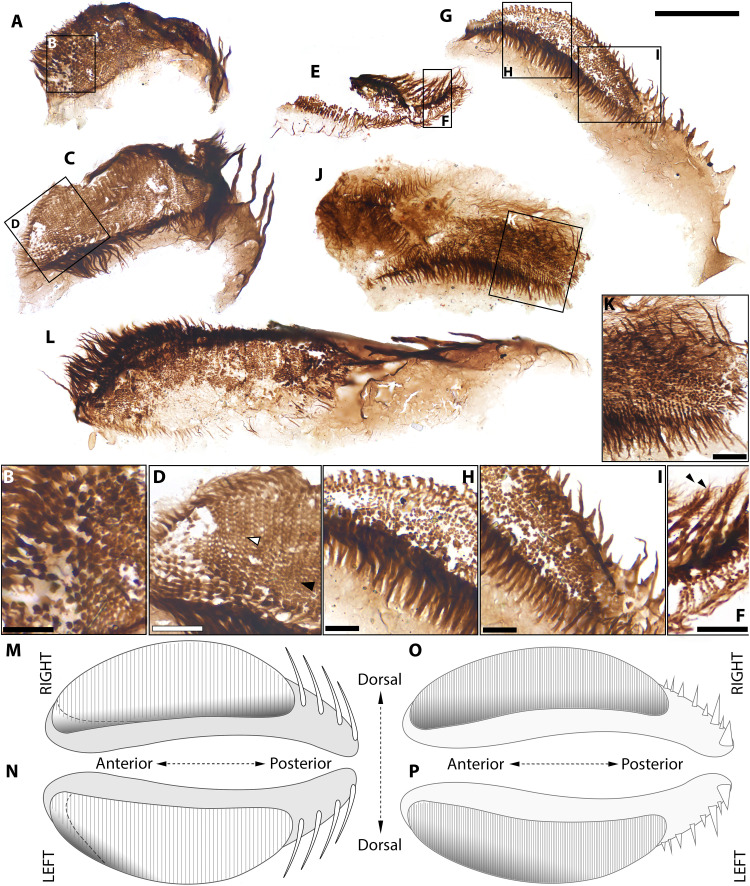
Crustacean molars from the Bright Angel biota. (**A** and **C**) Morphotype B molars [(A) left; (C) right]; (C) is reflected for ease of comparison. (**B** and **D**) Detail of boxed areas in (A) and (C), showing oblique spinose ornament and marginal fringe, and asymmetric gradation from circlet-like (black arrowhead) to tuberculate (white arrowhead) scales. (**E**) Partially preserved, probable morphotype A left molar [compare with setal pattern in (G) and (J)]. (**F**) Detail of boxed area in (E) showing marginal setae with lateral setules. (**G**) Morphotype A molar (right). (**H**) Detail of boxed area in (G) showing scaly lineations and marginal setal fringe. (**I**) Detail of boxed area in (G) showing posterior end of molar surface, showing marginal setae and spinose ornamentation. (**J**) Morphotype A molar (right). (**K**) Detail of scaly lineations. (**L**) Putative morphotype B molar (left). (**M** to **P**) Idealized reconstructions of morphotype A molars [(M) right molar; (N) left molar] and morphotype B molars [(O) right molar; (P) left molar], with shaded areas denoting placement of strongest setal fringes; anatomical coordinates are shown by accompanying arrows and text. Slide accession numbers and England Finder coordinates listed in data S1. Scale bars, 50 μm except in (B), (D), (F), (H), (I), and (K) (10 μm).

**Fig. 6.**
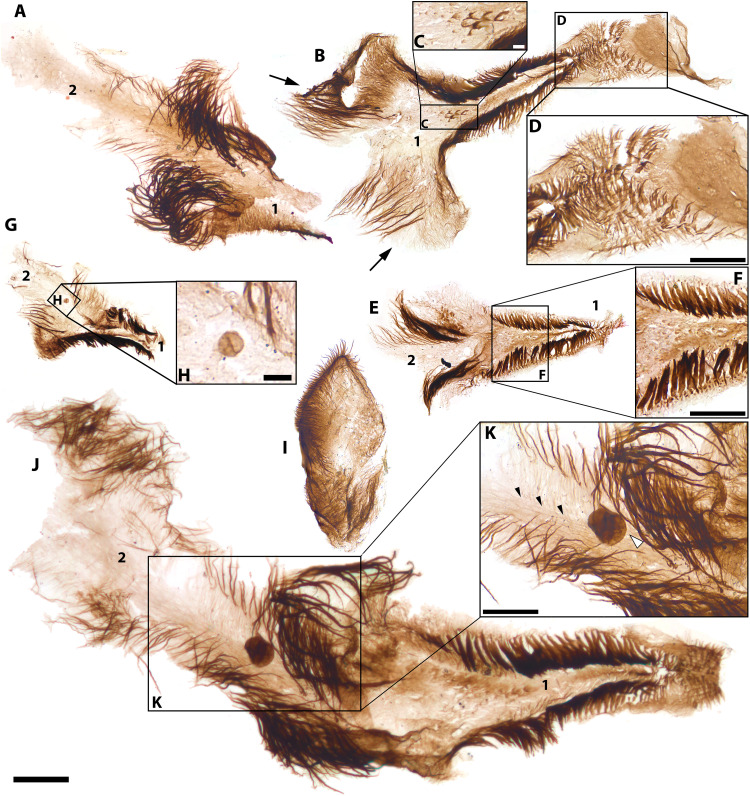
Crustacean sternal elements from the Bright Angel biota. Paragnath-bearing triangles are indicated by 1 s, and the successive posterior triangles by 2 s. (**A**) Posterior end of paragnath-bearing triangle and anterior section of successive triangle. (**B**) Complete triangle with laterally splayed paragnaths (black arrows). (**C**) Detail of scaly ornament of median cuticular strip in (B). (**D**) Detail of anterior setose surface in (B). (**E**) Complete paragnath-bearing triangle, with articulated anterior section of successive triangle. (**F**) Detail of boxed area in (E) showing scale-bearing cuticular strip and marginal setal fringes. (**G**) Articulated paragnath-bearing and partial posterior triangles defining median food groove. (**H**) Detail of boxed area in (G) showing sphaeromorphic acritarch adpressed onto the food groove cuticle. (**I**) Subtriangular cuticular lobe, representing possible setulose labrum. (**J**) Complete paragnath-bearing triangle and articulated, semi-complete posterior triangle. (**K**) Detail of boxed area in (J) showing spheroidal acritarch adpressed onto the food groove cuticle. Slide numbers and England Finder coordinates listed in data S1. Scale bars, 50 μm except in (C) and (H) (5 μm) and (D), (F), and (K) (25 μm).

**Fig. 7.**
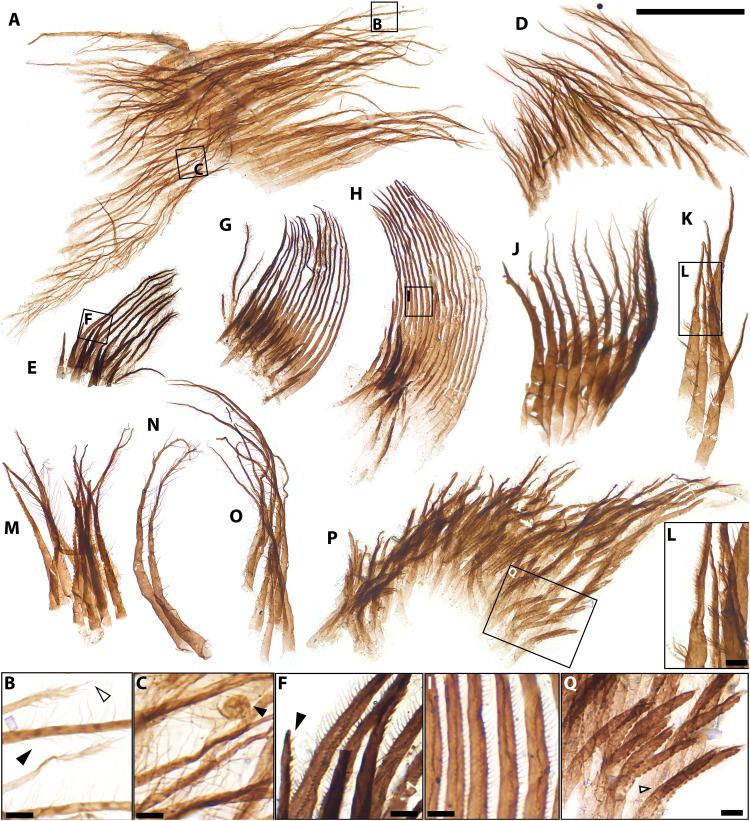
Setal arrays from the Bright Angel biota. (**A**) Pappose (bottom) and papposerrate (top) arrays. (**B**) Detail of boxed area in (A) showing distal terminations of papposerrate setae (white arrowhead) and biseriate laterals setules (black arrowhead). (**C**) Detail of boxed area of pappose array in (A), showing spheroidal acritarch adpressed onto the mesh of setules (black arrowhead). (**D**) Pappose array. (**E**, **G**, and **H**) Coplanar plumose armatures with accessory papposerrate setae. (**F**) Detail of boxed area in (E), showing plumose setae with biseriate lateral setules and distal termination of accessory seta (black arrowhead). (**I**) Detail of boxed area in (H) showing submicrometer-scale mesh defined by the coplanar plumose setae. (**J**) Serial array of setae bearing widely spaced lateral setules. (**K**) Cluster of setae bearing widely spaced tufts of setules and distal comb-like edge of sense setules. (**L**) Detail of boxed area in (K) showing comb-like edge and setal tufts. (**M**) Cluster of saw-toothed setae. (**N** and **O**) Clusters of setae bearing widely spaced pairs of setules. (**P**) Arcuate array of saw-toothed setae. (**Q**) Detail of boxed area in (P) showing split setal terminations, saw-toothed setal edges, and opposite edge bearing fine setules (black arrowhead). Slide numbers and England Finder coordinates listed in data S1. Scale bars, 100 μm except (B), (C), (F), and (I) (5 μm), and (L), Q (10 μm).

We identified crustaceans based on their lineated scaly molars (*55*) showing multiple diagnostic similarities to those of extant branchiopods: a simple crescentic or D-shaped outline (Fig. 5, A, C, G, J, and L), numerous densely spaced scale rows (Fig. 5, B, D, H, and I), unilateral variation in scale size and morphology, and an oblique marginal field of differentiated setae or spines (Fig. 5, B to D), compare with (*55*–*57*). This diagnostic trait combination (*58*) argues against alternative branchiocarid, hymenocarine, or waptiid affinities and permits direct comparisons with previously reported Cambrian branchiopod–type SCFs (*24*, *58*, *59*). The diagnostic BAF molars co-occur with triangular sternal elements and setal arrays showing detailed similarities to known crustacean counterparts (Figs. 6 and 7 and figs. S9 and S10). These elements complement the molars to form a taxonomically and functionally coherent complex, suggesting a shared set of branchiopod producers (see section “Small carbonaceous fossils, Crustaceans”).

**Fig. 8.**
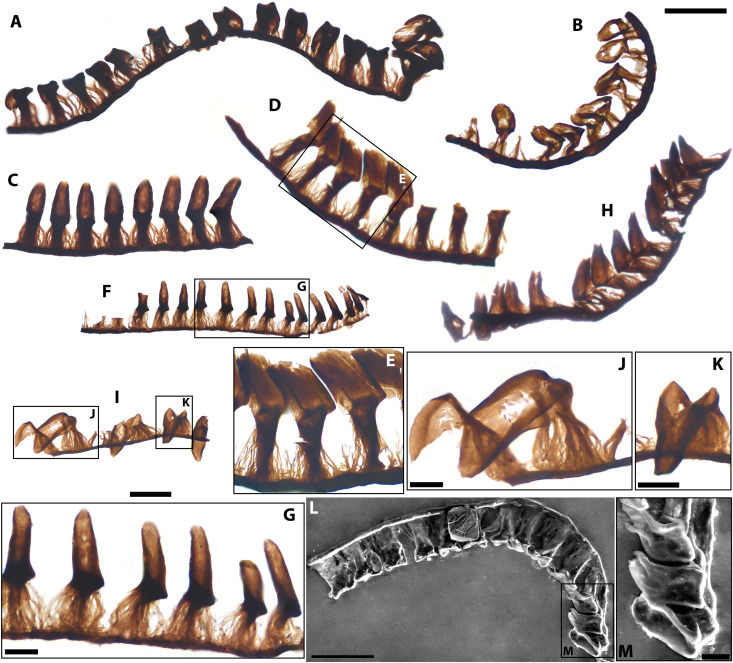
Molluscan radulae from the Bright Angel biota. (**A**) Series of shovel-shaped teeth displaying an element of bilateral symmetry. (**B**) Recurved partial series of shovel-shaped teeth. (**C**) Partial series of peg-shaped teeth. (**D**) Partial series showing teeth with marked terminal wear. (**E**) Detail of boxed area in (D). (**F**) Partial series of peg-like teeth. (**G**) Detail of teeth in boxed area in (F) showing low to moderate terminal wear. (**H**) Partial series of peg-like teeth displaying terminal wear. (**I**) Partial series of shovel-like teeth. (**J** and **K**) Details of boxed areas in (I), showing concave dorsal surfaces of teeth. (**L**) Scanning electron microscopy image of a specimen with shovel-like teeth. (**M**) Detail of boxed area in (L) showing concave tooth surfaces. Slide numbers and England Finder coordinates listed in data S1. Scale bars, 50 μm except in (E) (25 μm), (G), (J), (K), and M (10 μm).

Radular elements (i.e., parts of the ribbon-like, toothed feeding apparatus of molluscs; Fig. 8) show a diagnostic combination of a microstructure of dense parallel fibers, denoting microvillar secretion, and longitudinal series of protruding teeth (*60*, *61*). Although the microvillar construction of sclerotized body parts is a probable synapomorphy of Lophotrochozoa, extensive belt-like series of rigid teeth joined by longitudinal supports are characteristic of molluscan radulae (*60*, *61*). In addition, the BAF radulae are directly comparable to more extensively articulated SCFs from the early Cambrian Mahto Formation of Alberta, Canada, which display the diagnostic molluscan arrangement of multiple, closely spaced en echelon rows of teeth disposed in bilaterally symmetrical fashion (*60*); see section “Small carbonaceous fossils; Molluscs.” This morphology argues against alternative annelid or gnathiferan associations (*60*).

Besides elements with diagnostic traits of particular animal phyla, the BAF SCFs include more fragmentary and taxonomically problematic remains. Among them are cuticles with subcircular, irregularly distributed tubercles potentially belonging to bradoriid arthropods, previously reported from other Cambrian SCF shelf biotas (*26*). In addition, the BAF biota comprises 113 fossils of microbial origin. Non-metazoan elements include ~103 spheroidal acritarchs between ~4 and 300 μm in diameter, and 10 aggregates of cyanobacterial-grade filaments (see the Supplementary Materials, fig. S3). The presence of probable microbial photosynthesizers is consistent with a depositional environment within the photic zone. We did not find previously reported “cryptospore-like” palynomorphs, such as tetrads and dyads from the eastern BAF (*37*), in the newly sampled, more oceanward localities (Fig. 1C).

Together, the Bright Angel SCFs span a wider size range than their previously described ~10^1^- to 10^2^-μm scale Cambrian counterparts (*48*): specimens range from ~4-μm-wide microorganisms up to macroscopic, exceptionally articulated ~3-mm-long metazoan specimens (Fig. 2A, compare with 7H). Similarly, the Bright Angel SCFs vary greatly in their degree of articulation. The recovered molluscan radulae are invariably incomplete and isolated from other body parts (Fig. 8). Crustacean elements display more variable degrees of articulation, ranging from semi-isolated setae to interlocked sternal elements and complete filter plates (Figs. 5 to 7 and figs. S9 and S10). However, the greatest variation is found among the BAF priapulid SCFs, which range from isolated sclerites to millimeter-scale articulated body parts (Figs. 2 to 4 and figs. S4 to S8). Together, partly articulated elements comprise ~37% of all SCFs recovered in the BAF (data S1).

#### Priapulids

*Description*. The BAF priapulid pharyngeal teeth form a morphological continuum of V- to U-shaped sclerites, ranging in length from ~10 to ~120 μm across different specimens (Figs. 2 to 4 and figs. S4 to S8). Exceptionally complete and articulated specimens (Figs. 2 to 4) permit the attribution of all priapulid SCFs recovered from the BAF (*N* = 967) to a single taxon, *Kraytdraco spectatus* gen. et sp. nov (Figs. 2 to 4 and figs. S4 to S8; see Supplementary Text–Systematic paleontology). This attribution is justified by the shared diagnostic features, co-occurrence, and mutual overlap of pharyngeal sections bearing distinct tooth types, which enable the reconstruction of a biologically coherent element zonation pattern. As a result, the Bright Angel pharyngeal teeth can be classified as “distal” (i.e., anteriormost when everted) or “proximal” (i.e., posteriormost when everted) in relation to their position along the everted pharynx (*51*, *52*).

The most extensively articulated priapulid fragments from the BAF are up to *~*3 mm long and comprise several hundred individual teeth (Figs. 2A and 3L). These specimens preserve tracts of the eversible pharynx in its inverted, “at rest” configuration (*49*, *52*), as evinced by the medially rather than outwardly pointing orientation of the pharyngeal teeth (Figs. 2, A and Q, and 3, D, E, I, and L) (*49*, *52*) and their recessed position relative to surrounding sections of polygonally patterned, ensheathing cuticle (Fig. 3, D, E, and L, and fig. S4, H, N, and O). Along its length, *Kraytdraco*’s pharynx bears two regions separated by a tapering transitional zone. One region comprises a “narrow” tract (proximal in the everted pharynx; e.g., Fig. 2, A, K, N, and Q) that in the best-preserved specimen appears to flare at the top of the inverted pharynx and to extend into a funnel-like constriction toward the bottom (Fig. 2A). The other region consists of a wider tract (distal in the everted pharynx; e.g., Fig. 3, E, L, and Q, and fig. S4 K, N, and O) at least one-third broader than its narrow counterpart (Fig. 3L, compare with 3E). The presence of a rounded termination to the “wide” tract [which appears “blind” or “closed” when seen in lateral view; Fig. 3Q, compare with e.g., fig. 3 of (*53*)] is inferred to represent the distalmost tip of the everted pharynx [(*49*, *51*, *52*) movie S1].

From this distal tip to the transitional zone between the narrow and wide tracts, the pharynx bears a continuum of U-shaped to subtriangular teeth (Fig. 3, A, C, E, and G to R, and fig. S4, A to C, G, J, and K to O) with a tongue-shaped basal pad, up to ~five times the length of the more sclerotized arch (Fig. 4, K to P, and fig. S5, C to K). The arch bears a total of six to eight spine-like denticles arranged symmetrically on the two sides of a subconical prong, taller and more robust than the flanking denticles. The denticles are frequently interspersed with thin, unbranched filaments up to ~3.5 times their length [Fig. 4, K, L, and O, and figs. S4J, S5 (C, E, G, H, J, and K), and S6 (H and I)].

In the transitional zone between the wide and narrow pharyngeal tracts, the U-shaped teeth show progressively shorter spine-like denticles, and grade into increasingly less sclerotized elements showing a subdued multicuspidate prong, a basal pad with marked transverse rows of comb-like projections, and an arch bearing dense filamentous denticles with no spine-like counterparts (Fig. 4, A to C and G, compare with 4, I, K, to O; Fig. 3, G, J to M; and fig. S5, C to E, compare with A and B). These filamentous denticles often extend beyond the tooth apex and display up to seven to eight orders of delicate bifurcating tendrils or consecutive side branches (e.g., Fig. 4, C and J, compare with Fig. 2T), thinning distally. The denticles closest to the base of each tooth splinter basally into a bush-like architecture (e.g., Fig. 4G), whereas closer to the apex of the tooth the denticles show a distinct main “shaft” with fine side branches (Fig. 4C and fig. S7N).

The teeth occupying the transitional zone grade smoothly into the triangular sclerites of the narrow tract (Fig. 4, B and C, compare with Fig. 2, S to U). Distally, the narrow tract bears broadly equilateral teeth similar to those occupying the proximal-most transitional zone, but with a more marked multicuspidate prong (Fig. 2, S to U) and sparser denticles with progressively fewer orders of bifurcations apically (down to zero near the base of the prong). More proximally in the narrow tract, the apical denticles of this tooth type show blunt, tubercle-like lateral projections in place of the filamentous side branches (Fig. 2M and figs. S6, D and E, and S7H). The proximal-most region of the narrow tract is occupied by elements with a more elongate to falciform arch and a simpler, unicuspidate prong (Fig. 2, B and L, compare with Fig. 2J and fig. S7, F and I). In these sclerites, the branching filamentous denticles become increasingly restricted to the basalmost part of the arch. By contrast, the apical portion of the arch bears unbranched filamentous denticles with a thickened, club-like termination (Fig. 2, C, G, L, and J, and figs. S6, C and K, and S7, F, G, I, and J).

Isolated sclerites in the Bright Angel assemblage are interpreted as probable priapulid introvert hooks (Fig. 2, E, F, and H, and fig. S7, E and C), recognizable by their high aspect ratio, curved margin, denticulate arch, and greater length (up to ~250 μm) compared to co-occurring teeth (*49*, *51*). The Bright Angel hooks resemble similarly sickle-shaped counterparts in the Burgess Shale priapulid *Ottoia* (*51*, *52*), and likewise bear two parallel rows of denticles along their concave midline (fig. S7C and Fig. 2, E and H). Unlike those of *Ottoia*, their basal denticles bear slender side branches, comparable to those adorning the teeth of *Kraytdraco*’s narrow tract; moreover, their falciform profile resembles that of the most proximal teeth in the narrow tract (Fig. 2I and fig. S7D). On the basis of this shared diagnostic trait and a lack of other recognizably distinct priapulid elements in the BAF, the hooks are tentatively attributed to *Kraytdraco*. Of these hooks (*N* = 35), 25 are physically associated with unornamented, smooth-walled conical sclerites with a blunt termination, somewhat reminiscent in size and shape to the hollow scalids housing adhesive or sensory tubuli in extant [e.g. (*53*)] and fossil (*22*, *62*, *63*) priapulomorphs; however, no apical opening is clearly discernible in the *Kraytdraco* specimens [Fig. 2, E and F, and fig. S7E, compare with (*53*), fig. 10]. Other SCFs similarly lacking a distinct arch, prong, or basal pad characteristic of pharyngeal teeth, but showing a basally inflated surface and denticulate ridges running along their apices, may record trunk sclerites like those known from extant priapulids [fig. S5, A and B, compare with (*49*, *64*) fig. 3C].

*Functional morphology*. The delicate submicrometer-scale branching tendrils and weakly sclerotized construction of *Kraytdraco*’s teeth would have been poorly suited for macropredation—conspicuously unlike the cuspidate (dagger-like) counterparts found in other Cambrian priapulids and the extant Halicryptidae and Priapulidae (*49*, *50*). The pharynx of the deposit feeding and/or detritivorous priapulid *Tubiluchus* (*65*–*67*) offers a more convincing modern counterpart. The pharynx of *Tubiluchus* bears cuticular teeth with a pectinate (comb-like) substructure. Posteriorly, these teeth grade into fimbrillae, which consist of semicircular cuticular flaps with fine bristles along the distal margin. Although the fimbrillar bristles of *Tubiluchus* are unbranched, they recall the filaments of *Kraytdraco* in their delicate construction, flexibility, and extension relative to their supporting arch; compare with fig. 7D of (*68*).

These morphological similarities suggest a form of selective detritivory or deposit feeding in *Kraytdraco*. Like those on the pectinate teeth of *Tubiluchus* (*68*), the more robust denticles on the distal teeth of *Kraytdraco* could have mobilized detritus by substrate raking or scraping (*65*, *66*). During pharyngeal inversion, the more proximal and fimbrillae-like branching filaments could then have swept and selectively trapped or “filtered” (*67*) food particles (*66*, *67*, *69*) into the bottleneck provided by the narrow tract. Even so, it is important to appreciate the order-of-magnitude larger tooth sizes of *Kraytdraco* relative to the ~5- to 10-μm-long teeth of the millimeter-long, meiofaunal *Tubiluchus* (*65*, *68*), and the proportionately wider spaces between their filamentous denticles [Figs. 2 and 4, compare with (*68*), fig. 7D and (*65*) fig. 7]. These tooth size differences most likely correspond to similar differences in overall body size. Summing the length of the contiguous and partly overlapping pharyngeal sections of *Kraytdraco* (Fig. 2, A and Q, compare with Fig. 3, E, L, and Q) yields a conservative length estimate in the 3- to 4-mm range for the entire pharynx. Applying the same approximate scaling relationships of pharynx and body length observed in *Ottoia*, where similarly to most modern priapulids (*49*, *53*, *64*, *68*, *70*) and palaeoscolecids (*71*, *72*) the everted pharynx measures ~10 to 20% of total body length [(*52*), plates 3 (fig. 3) and 11 (fig. 2)], yields a size in the 1.5- to 4.0-cm range for *Kraytdraco* specimens recorded by partially articulated tracts. However, these specimens likely record juveniles given the smaller sizes of their constituent teeth compared to isolated counterparts (e.g., Fig. 2A, compare with J, M, and U), suggesting greater adult body lengths. Whereas modern meiofaunal priapulids likely feed on interstitial bacteria (*65*–*67*), based on its tooth and body sizes, *Kraytdraco* would have exploited larger food items, possibly encompassing eukaryotic phytobenthos, meiofauna, and/or microdetritus.

The filamentous extensions of *Kraytdraco*’s proximal teeth might also evoke a suspension feeding role. However, this lifestyle has no known ecological equivalents among priapulids. Furthermore, a suspensivorous lifestyle would leave unexplained the function of the robust denticles of the distal tract. The limited length of *Kraytdraco*’s branching denticles, similar to that of the accompanying tooth prongs, also contrasts with the extensive distal projections of invertebrates specialized in gathering suspended particles, as seen in crustacean filter plates, lophophores, or annelid chaetae. Moreover, the small cross-sectional area to volume ratio of *Kraytdraco*’s everted pharynx would have lent itself poorly to the capture of suspended particles (Figs. 2, A and Q, and 3L, and movie S1).

#### Crustaceans

*Description*. Recognizably crustacean SCFs are represented in the Bright Angel biota by three element types: setal armatures (*N* = 72), cuticular triangles and lobes (*N* = 116), and mandibular molars (*N* = 13). We describe these elements in comparison with extant and Cambrian counterparts, adopting the alphanumeric classification system for spines, setae, and other arthropodan cuticular projections developed by Harvey and Butterfield (*58*) where applicable.

The BAF molars (Fig. 5 and fig. S9, B to H) are reliably identified by their ovoid to crescentic surfaces, ~125 to 220 μm long and up to ~50 μm wide, that bear transverse scaly lineations. The molar surfaces are bordered by setal fringes along their longitudinal margins (*55*, *58*). Two morphotypes are recognizable, likely recording distinct taxa given the diagnostic character of molar morphologies (*58*). In the first morphotype (morphotype A), the scales within each lineation are nearly continuous in size and shape across the width of the molar (Fig. 5, E to K, and fig. S9, B, D, and E), with a slightly negative size gradient from the concave to the convex margin. In the second—morphotype B—the scales grade asymmetrically from larger, robust tuberculate protrusions near one margin to shallower, closely spaced “circlets” near the other [Fig. 5, A to D, compare with (*73*) pl. 5]. A field of spinose [A5 (*58*)] setae, merging laterally into the marginal setal fringe, extends obliquely along the posterior margin (e.g., Fig. 5D).

Unlike previously described branchiopod-type specimens from Cambrian SCF biotas (*24*, *58*, *59*), two molars attributable to morphotype A have a conspicuous incisor process extending posteriorly to the lineated molar surface. The incisor edge bears up to 13 to 14 cuspidate teeth of irregularly varying length and thickness (Fig. 5G and fig. S9F). Morphotype B specimens have a similar incisor process, which bears a series of at least four thinner, more elongate spines supporting minute lateral spinules distally (Fig. 5, A and C, compare with L). An incisor process is diagnostically absent in branchiopods crownward of the upper Cambrian *Rehbachiella* (*58*, *74*), and either not visible (*58*) or clearly absent (*24*) in previously recognized anostracan-type Cambrian SCFs (*58*). This trait suggests a more basal placement for the Bright Angel specimens than for other Cambrian SCF counterparts—potentially one in the branchiopod stem group (*58*, *74*).

Two distinct configurations are expressed by different specimens of the morphotype A and B molars: one bearing the most marked setal fringe, with robust setae often bearing fine setular outgrowths [longest distally, compare with C15-16 (*58*)], along its less curved or “convex” margin (Fig. 5, A and L, and fig. S9, G and H), and the other bearing this robust setal fringe along its more curved or “concave” margin (Fig. 5, C, D, G, H, and J, and fig. S9, D and E). By comparison with known extant and Cambrian branchiopods, the arrangements of setal fringes permit the identification of right and left molars in the Bright Angel assemblage. The right molars bear their most robust setal fringe on the ventral (concave) margin, whereas the left molars bear it on their dorsal (convex) margin (*55*, *58*). The consistent scaling relationship between molar surface and body length in living crustaceans [see fig. S2 of (*59*)] indicate that the ~125- to 220-μm-long Bright Angel specimens probably belonged to ~10- to 15-mm-long animals, somewhat smaller than other probable Cambrian branchiopods from the Deadwood Formation SCF assemblage of Saskatchewan, Canada, and the Mount Cap Formation and Mount Clark Formation SCF assemblages of the Northwest Territories, Canada (*24*, *58*, *59*).

The Bright Angel molars co-occur with cuticular triangles (Fig. 6, A to G, J, and K, and figs. S9, I to Q). These triangles comprise 115 bilaterally symmetrical, isosceles-shaped elements ~200 to 500 μm long and up to ~150 μm wide. Their tapering outlines, basally flaring and somewhat concave median strip of cuticle (Fig. 6, C, F, and K), and parallel fringes of robust distally blunt setae [compare with A1 (*58*)] on their longitudinal margins identify the cuticular triangles as crustacean sternal elements, forming a continuous “food grove” tapering anteriorly (*75*–*77*). Subtle variation in the convexity of the food groove and the robustness of the fringing setae across different specimens (fig. S9, A, K, and Q, compare with S9, L, O, and P) may be taxonomic in origin, and parallel the differences in cuticular ornamentation between the co-occurring morphotype A and B molars. Irrespective of this morphological variability, the parallel setal fringes and median cuticular strip of the BAF triangles are similar to those of sternal elements in both crown-group (notostracan) branchiopods [see fig. 24 in (*75*) and figs. 77 and 102 in (*78*)] and *Rehbachiella* (*77*). However, unlike equivalent paired triangles from the Mount Clark assemblage (*58*), the Bright Angel elements are not divided by a median cleft into bilaterally symmetrical halves. The same undivided condition is observed in the sternal elements of *Rehbachiella*, consistent with a closer affinity to this stem-group taxon (*58*, *77*). Like the anteriormost sternal element of *Rehbachiella* (*77*) and notostracans [see fig. 24 in (*75*)], most of the Bright Angel sternal triangles extend basally into two lateral outgrowths, each bearing dense fields of pappose [compare with C19 (*58*)] setae (e.g., Fig. 6K) along its distal margins. These outgrowths are recognizable as paragnaths—foliose cuticular protrusions lying behind the mandibles—based on their lobate shape and basal position on the cuticular triangles [e.g., Fig. 6, B and E, and fig. S9, O and P; compare with pl. 27:4 of (*77*) and fig. 24 of (*75*)].

Multiple specimens of paragnath-bearing elements articulate posteriorly with a second, contiguous cuticular triangle terminating between the bases of the paragnaths (Fig. 6, A, B, E, G, and J). Compared to the paragnath-bearing element, the second triangle in these “articulated” specimens consistently has a less markedly tapering outline than the first, including fine striations instead of minute scales adorning its cuticular strip (e.g., Fig. 6K, compare with C), lateral fringes of more delicate and thinner setae [compare with A12, C20 (*58*)], and no visible paragnaths. Together, the paragnath-bearing triangle and its posterior counterpart define a continuous groove flanked by pappose setal fields and fringes, presumably spanning at least two consecutive sternites [see pl. 27:4 of (*77*)]. The groove terminates anteriorly into a broad, pauldron-like cuticular lobe covered by dense subconical setules [compare with F2 (*58*)] (Fig. 6D), which may have floored the oral opening and/or the anteriormost esophagus (*58*, *75*, *77*, *78*). In *Rehbachiella* and extant anostracans, the oral termination of the food groove is delimited by an overlying labrum (i.e., upper lip). The labrum, which remains unknown in other Cambrian SCF crustaceans (*58*), is potentially recorded in the Bright Angel assemblage by a distinctive unpaired, bilaterally symmetrical, and subtriangular cuticular lobe covered by short setules [F1 (*58*)] around its distal margin [Fig. 6I; compare with (*75*, *77*, *78*)].

After the sternal triangles, the second most abundant category of Bright Angel SCFs attributable to crustaceans are setal arrays (*N* = 72). The most distinctive are coplanar armatures with arcuate distal outlines (*N* = 18), each comprising ~20 to 50 plumose setae [D1 (*58*)] up to ~330 μm long that bear thin, biserially arranged setules spaced at regular intervals of ~1 μm or less (Fig. 7, E to I, compare with fig. S10, A to E, G, H, J, K, and M to O). The close spacing of the setules in strictly opposite series, the coplanar arrangement of their supporting setae, and the distal curvature of the arrays identify these SCFs as filter plates (*58*, *59*, *75*, *79*, *80*). Their characteristic arcuate shape and co-occurrence with the taxonomically informative molars suggest that these filter plates belonged to the thoracic appendages of branchiopods (*24*, *58*, *75*, *76*). A branchiopod attribution is further justified by the presence of accessory setal arrays (Fig. 7, E to H, and fig. S10, C to E and M to O) preserved together with the filter plates and flanking them unilaterally (*58*) as occurs in modern members of this class (*58*, *75*). The accessory arrays comprise papposerrate setae [C5 (*58*)] that proximally bear long, delicate setules (Fig. 7, E to H, and fig. S10, C to E and M to O) and distally bear serrate, uniserially arranged denticles (Fig. 7F and fig. S10, N and J). These setae may reach up to two-thirds of the length of their flanking filter plates.

Other arcuate setal arrays, found without associated filter plates (*N* = 45), consist of pappose [compare with C19 (*58*)] to papposerrate setae bearing fine biseriate lateral setules (Fig. 7, A to D and P, and fig. S10P). A subset of pappose arrays are “saw-toothed” forms that have blunt serrations on one edge, down to the base of the seta, and distal bifurcations with one branch setulose and the other continuing the saw-toothed ornament (Fig. 7, P, Q, and M). Unlike in similar saw-toothed setae (C14 and B1 to B3) from the early Cambrian Mount Clark biota (*58*), the Bright Angel setules appear to insert only on one edge of the shaft, opposite to the serrations.

Unlike the Mount Clark and Deadwood crustacean SCFs (*24*, *58*, *59*, *79*), the Bright Angel assemblage also comprises nine arrays of hollow setae bearing widely spaced pairs of setules (Fig. 7, J, N, and O, and fig. S10F), which become finer, denser, and approximately 75% shorter in the distalmost part of the shaft (Fig. 7O and fig. S10F). Other similar Bright Angel setae bear widely spaced tufts of three to nine setae each (Fig. 7, K to L, and fig. S10, I and L) and a “finger”-like distal termination bearing a weakly recurved, uniseriate comb-like edge. The “comb” consists of short (~3 μm) densely spaced setules. Both of these array types are always disassociated from filter plates or their accessory setae, supporting a different position in life.

*Functional morphology*. The BAF crustacean molars, setal arrays, and sternal triangles are not found articulated together. Thus, conclusive attribution of particular setal types or sternal elements to the individual taxa represented by morphotype A and B molars would be premature. Nonetheless, all these element types find morphological counterparts among known stem and/or crown-group branchiopods. In addition, they express distinct but complementary adaptations to the capture and processing of a similar range of particulate food. These features invite the reconstruction of a feeding complex coherent in terms of its phylogenetic signal, functional morphology, and target food items.

By comparison with modern mandibulates, conspicuous spinose and tooth-like gnathal protrusions on the Bright Angel molars may represent an adaptation for holding onto and/or macerating relatively large food items (*55*, *73*). Elongated incisor spines, sometimes with barbed morphologies (compare with Fig. 5C), are common among carnivorous and omnivorous mandibulates [e.g. fig. 3A in (*55*); figs. 1E and 6, A to D in (*81*); and fig. 2 in (*82*, *83*)]. Moreover, the asymmetric gradation from tuberculate to shallower, circlet-like scales of morphotype B (e.g., Fig. 5, A to D) is reminiscent of the pattern in the extant suspension feeding but facultatively predatory anostracans *Branchinecta cornigera* and *Branchinecta paludosa* [compare with (*73*), pl. 5 and 6]. Although these modern branchiopods lack gnathal edges with acuminate teeth or spines, their scales are less uniform and more robust than those of obligate particle feeders [e.g. (*73*), pl. 1 and 2; compare with (*58*)], suggesting similar mixed trophic habits in their Bright Angel counterparts. In this light, the more diminutive, less asymmetrical scales of morphotype A molars may suggest a lifestyle shifted more toward herbivory. Accordingly, counterparts to their short, spine-like, subconical incisor teeth are phylogenetically widespread among non-crustacean [e.g. (*55*), fig. 1] and crustacean [e.g. (*83*), figs. 3 and 2A and fig. 2C in (*24*)] mandibulate herbivores, where they may perform a cracking action on recalcitrant food items and are typically less elongated than counterparts in predatory taxa (*83*). Irrespective of these specializations in incisor and scale structures, the overall morphology of both types of BAF molars supports principally planktotrophic diets. Their elongated, finely ornamented surfaces covered by dense scaly lineations consistently denote microphagous suspension feeding in modern counterparts, even those displaying occasional carnivory (*55*, *73*).

The largely particle-feeding feeding lifestyle suggested by the Bright Angel molars is coherent with the morphology of co-occurring sternal triangles. As in modern branchiopods, the paragnaths and laterally fringed sternites of the thoracic food groove would have defined a continuous channel. Together with the overlying labrum, this structure would have conveyed suspended food particles to the mouth, where they would have been ground by the molars (*75*–*77*). Within their sternal food groove, at least three specimens of BAF sternal triangles (Fig. 6, G, H, J and K, and fig. S9, I and J) preserve small (4 to 16 μm wide) and most likely planktonic (*84*, *85*) sphaeromorphic acritarchs, not found associated with non-crustacean Bright Angel metazoan remains. The position of these organic-walled microfossils, medial to the paragnaths and setal fringes that would have directed food to the mouth (*75*–*77*), is consistent with a trophic association, in keeping with the morphology of the co-occurring molars.

The particle channeling and triturating complex of the molars and sternal elements would have been functionally complementary to the BAF setal arrays. The filter plates on the thoracic assemblages of modern branchiopods consistently fulfil suspension feeding roles (*24*, *58*, *75*, *76*). Similarly, in the case of the BAF crustaceans, the suspended particles could have been gathered from the water column by the densely spaced plumose setae. The diverse co-occurring setal arrays would have been suitable for subsequent size-sorting, screening, and transfer of captured particles for grinding by the molars (*58*, *75*, *76*). For instance, the comb-like structure of setae bearing comparatively robust and sparse setules (Fig. 7, J to L, N, and O, and fig. S10, F, I, and L) suggest a screening function [compare with (*86*), fig. 7], potentially paralleled by similar maxillary setae in *Rehbachiella* [compare with (*77*), pl. 19, fig. 6]. Meanwhile, the small size of the saw-toothed setae and their association with filter plates suggest a complementary role in trapping food particles or clearing the food groove (*58*) rather than substrate scraping. A similar particle-handling function for the pappose arrays is consistent with the minute (~6-μm-wide) acritarch found on one of their submicrometer-scale meshes (Fig. 7C): Its spheroidal morphology and small (<10 μm) size support phytoplanktonic origins (*84*). Together, the diverse complement of setal armatures and filter plates of the Bright Angel SCFs, absent in *Rehbachiella* (*58*, *77*), suggests a distinct feeding mode directly comparable to that of crown-group branchiopods (*75*, *76*) despite the retention of a somewhat plesiomorphic, incisor-bearing molar architecture.

Unlike the Deadwood, Mount Clark, and Mount Cap branchiopods (*24*, *58*, *59*, *79*), the Bright Angel arthropod SCFs lack any associated club-shaped, spinose, or toothed plates and setal armatures for substrate processing despite the considerable robustness and preservation potential of these elements compared to delicate plumose or pappose armatures (*58*, *59*). This absence argues against facultative substrate scraping in combination with suspension feeding (*24*, *58*, *73*).

#### Molluscs

*Description*. Newly recovered radulae from the BAF (Fig. 1C) comprise 11 elements ~150 to ~450 μm long, conspicuously darker, more opaque, and more three dimensional than other co-occurring SCFs (Fig. 8). All consist of a series of shovel- or peg-like “teeth” numbering 3 to 16 per (incomplete) specimen. The ~15- 35-μm-long and ~5- 25-μm-wide teeth tend to imbricate longitudinally. They are joined basally by an elongate rod, subcylindrical in cross section (Fig. 8, A, B, and L, compare with C and F). Each tooth is boot-shaped and comprises a distal “sole” and a basal “ankle.” The ankle consists of a fibrous constriction ~20 to 35 μm long and ~10 to 20 μm wide, joining the sole to the basal longitudinal rod. The sole articulates with the ankle at a ~90° to 130° angle. It is wedge-shaped and apically flattened, with a distal end or “toe” occasionally displaying blunt rounded terminations denoting probable terminal wear [Fig. 8E, compare with J and G; compare with (*60*)] and expanding posteriorly into a protuberant “heel.” The sole’s upper surface is weakly concave, with a broad longitudinal groove (Fig. 8, J, K, and M). The width and tapering of the sole vary across different specimens, permitting the recognition of two different morphotypes potentially corresponding to distinct tooth rows in the radular apparatus: a shovel-like form with a broad, nearly ovate sole (e.g. Fig. 8, A, B, and L), and a peg-like form distinguished by its higher aspect ratio and slender morphology (e.g., Fig. 8, C and F).

With their rounded tooth terminations and imbricate disposition along an extensive rod-like support, the Bright Angel specimens differ from both the discrete, bilaterally arranged, and more acuminate teeth of *Wiwaxia* and *Odontogriphus* from the Burgess Shale (*87*) and SCF radulae from the broadly coeval Earlie Formation, which have thinner spinose teeth arranged in a mirror-image pattern (*3*, *25*). By contrast, the Bright Angel radulae most closely resemble SCFs from the early Cambrian Mahto, Deadwood, Mount Cap faunas, and the Forteau Formation of (Newfoundland, Canada) faunas (*3*, *25*, *60*)—as well as previously reported fragmentary specimens from the eastern BAF [fig. S2, C and E; compare with (*3*, *37*)]—in having blunt wedge-like teeth with a fibrous ankle, arranged into a chain-like longitudinal series. The recovery of similar radular parts in the BAF’s eastern exposures (Fig. 1C) suggests that molluscan producers occurred broadly from well-aerated mid-shelf settings to more estuarine environments to the east.

Like the Mahto radulae (*60*), the Bright Angel specimens have serially identical teeth within each row and do not show the median rhachidian tooth that likely characterized the common ancestors of Mollusca and of all radulate classes except the vermiform, shell-less, and secondarily reduced Aplacophora (*88*). Aplacophorans most likely form the sister-group of polyplacophorans (i.e., chitons), and both fossil and molecular evidence suggest that they originated from a chiton-like ancestor (*89*). Therefore, the aplacophoran-like morphology of the Bright Angel radulae (and their Mahto counterparts) may be interpreted as derived, aligning these SCFs with total-group Aplacophora rather than stem-group chitons [compare with (*60*)].

*Functional morphology*. The extensive longitudinal arrangement, differentiated rows of recurved peg to boot-like, and distally blunted radular teeth of the Bright Angel radulae (Fig. 8) argue against a simple raking action, as inferred for the generalist Burgess Shale taxa *Wiwaxia* and *Odontogriphus* [compare with (*87*); see (*60*)]. These two lophotrochozoans have short transverse rakes of scoop-like and acuminate teeth, likely adapted for nonselective grazing or detritivory on soft substrates (*87*, *90*). By contrast, the putative terminal wear, extensive chain-like configuration, and close similarities to known multi-echelon complexes (*60*) of the Bright Angel radulae suggest a more sophisticated conveyor belt–like mechanism akin to that of patelliform gastropods and chitons (*60*, *91*), where successive rows of recurved teeth are drawn anteroposteriorly to abrade or scrape algae or microorganisms from firm substrates. This feeding mode is consistent with the co-occurrence of the BAF radulae with probable microbial mat–derived filaments (e.g., figs. S2, A and B, and S3, H to J), as well as the record of Cambrian traces of radular scraping found on microbial substrates and associated with molluscan locomotory traces (*92*).

### Paleoecological indicators

The functional morphologies represented by the Bright Angel SCFs are complemented by diverse indicators of the levels and autecological correlates of animal activity in the broader paleoecosystem. The most informative of such proxies are provided by the BAF’s ichnological record (*37*).

Ichnofossils are comparatively abundant, widespread, and well-characterized in the BAF. Animal traces have been reported in approximately 86% of decimeter-scale intervals in composite sections from the western BAF in the Grand Canyon of ~90 m measured from four outcrops; and in ~96% from eastern counterparts, of ~282 m measured from seven outcrops [fig. S11; see (*39*), figs. 15 and 16, and appendices 1.1 to 2.5]. Together, these measured sections span the complete thickness of the BAF (fig. S11). Against the background of this relatively pervasive metazoan presence, the bioturbation intensities of studied sections of the BAF provide evidence for high levels of overall activity. Approximately 55% of intervals in both western and eastern composite BAF sections from the Grand Canyon show average bioturbation indices (BIs) ≥3, denoting moderate to complete bioturbation [see (*39*) and figs. 15 to 16]. High (BI = 4) to complete (BI = 6) bioturbation has been reported for *~*32% of intervals in western composite sections (*N* = 90 m) and for 34% in eastern ones (*N* = 282 m) [see (*39*) and figs. 15 and 16]. Among ichnofossil-bearing intervals, ~30% in the *Cruziana* ichnofacies and ~75% in the *Skolithos* ichnofacies show BIs ≥4; only ~30 and ~20%, respectively, have BIs below 3 [see (*39*) and fig. 21; compare with (*45*)].

**Fig. 9.**
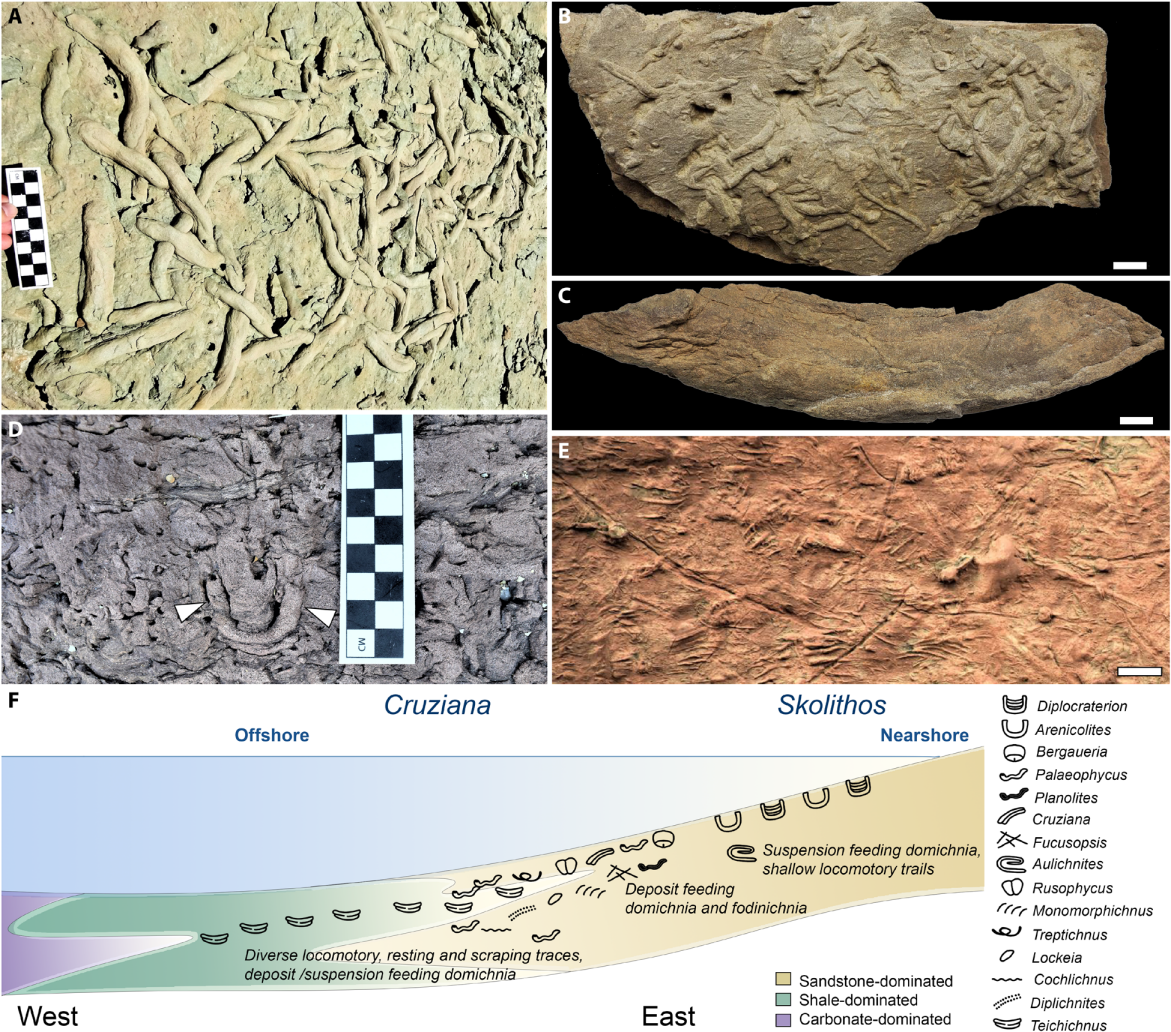
Representative ichnofossils and simplified ichnofacies distribution from the BAF in Grand Canyon National Park. (**A** and **B**) Bed soles with dense aggregates of *Palaeophycus*. (**C**) Isolated *Teichichnus*. (**D**) Lateral view of a U-shaped *Diplocraterion* in massive heavily bioturbated sandstone; white arrowheads denote opposite branches or “shafts” of the burrow. (**E**) Bed sole with abundant *Monomorphichnus*. (**F**) Schematic west to east cross section of BAF paleoenvironments, showing the *Skolithos* and *Cruziana* ichnofacies with ichnofossil distribution, depositional setting, predominant lithologies (color-coded legend), and inferred tracemaker behaviors after Miller (*39*) Scale bars, (B, C, and E) 10 mm.

In line with the conspicuous particle feeding adaptations of the BAF’s SCFs (Figs. 2 to 4, 6, and 7), the dominance of its ichnofossil record by traces attributable to deposit or suspension feeding worms suggests a thriving plankton and/or microphytobenthos [Fig. 9 and fig. S3 (*37*, *39*)] and high availability of both suspended and settled organic particles (*37*, *39*, *44*). The most common ichnogenus in the BAF’s *Cruziana* ichnofacies is *Teichichnus*, an arcuate spreiten-bearing burrow (Fig. 9C) likely produced by the activity of vermiform, infaunal suspension or deposit feeders (*93*). In the BAF, *~*80% of its reported occurrences map onto moderately to fully bioturbated intervals (BIs = 3 to 6); heavily to completely bioturbated intervals (BIs = 4 to 6) correspond to *~*31% of occurrences [see (*39*) and fig. 18, compare with fig. 37B]. The second-most common ichnogenus in the *Cruziana* ichnofacies, *Palaeophycus* (*37*, *39*, *44*), consists of subhorizontal, passively filled lined burrows (Fig. 9, D and E) generally interpreted as domichnia of suspension feeding worms (*94*). These burrows also occur at high densities (*37*) in the BAF [e.g., Fig. 9, A to D]: ~32% of reported local occurrences of *Palaeophycus* correspond to BIs of 4 to 6, and 68% to intervals at least moderately bioturbated [BI ≥3; see (*39*) and fig. 18]. Putative feeding traces morphologically similar to *Palaeophycus* are represented by the subhorizontal but unlined *Planolites* and *Fucusopsis*, which occur in moderately to very highly bioturbated intervals [BIs = 3 to 5; see (*39*) and fig. 18] and are thought to represent burrows by detritivorous or deposit feeding worms (*20*, *95*, *96*).

In its abundance of infaunal particle feeders and extent of heavily bioturbated horizons, the BAF contrasts with classic Cambrian BST-macrofossil Lagerstätten. Among them, the closest temporal, biogeographic, and paleoenvironmental counterpart to the BAF may be represented by the *~*507.5- to 506-Ma Spence Shale (Fig. 1A), which records inner to outer-shelf environments hypothesized to encompass shallower and better oxygenated conditions than those of other BST-macrofossil biotas (*18*). Nonetheless, the predominance of small burrows in the Spence Shale, its surficial (i.e., grazing, resting, and locomotory) traces, and its low bioturbation intensities hint at fundamentally more oxygen- or nutrient-limited conditions compared to the BAF (*20*, *21*). In the Spence Shale, reported ichnofabric indices range mostly between 1 and 3 (*19*, *20*); they reach values of 4 and 5 in only 4.5 and 2.9%, respectively, of the most intensely bioturbated section described (*19*). Pervasive millimeter- to centimeter-scale alternations of laminated and bioturbated intervals, a predominance of weakly to non-bioturbated horizons [up to 95.7% unbioturbated in the sections described by (*19*), compare with appendices 1.1 to 2.5 of (*39*)], and an absence of fully reworked substrates further support fluctuating oxygen levels insufficient to sustain protracted infaunal activity (*19*, *21*, *96*, *97*). Ichnofabric indices of 1 to 3, comparable to those dominating the Spence Shale, also appear to be most common in the similarly inner- to outer-shelf Kaili biota, although the overall intensity of local bioturbation has not been quantified (*16*). A similar predominance of low to moderate bioturbation (BIs 0 to 3) with sparse intervals of BI 4 to 5 characterizes studied sections of the early stage 4 Guanshan biota (fig. 2) (*98*). Yet, bioturbation in the Kaili, Guanshan, and Spence Shale biotas stands out as more common and intense than in other classic Cambrian macrofossil Lagerstätten (*4*, *11*, *12*, *20*), underscoring the BAF’s atypical bioturbation levels relative to these sites of “conventional” BST preservation.

Closer paleoecological counterparts to the BAF occur among other SCF localities. Mudstone horizons with thin silty sandstone interbeds from the upper Buen Formation, which has yielded earlier Cambrian (stages 3 to 4) SCFs (*26*), record relatively low-energy but well-oxygenated shelf settings sustaining high levels of bioturbation, although ichnofabrics have not been systematically quantified (*12*, *26*). The middle to late Cambrian Earlie/Deadwood succession of Saskatchewan, deposited in epicratonic waters of the Western Canada sedimentary basin (Fig. 1A), comprises highly variable facies and bioturbation intensities, with BIs mostly between 0 and 3; it also hosts fewer reported ichnogenera (*N* = 14) than the BAF [*N* = 21; see (*44*)], most of which record deposit feeding burrows (*99*, *100*). This record is consistent with stressed deltaic to nearshore environments through most of the succession (*96*, *97*, *99*, *100*), characterized by freshwater discharges, high-energy deposition, and limited nutrient and oxygen availabilities further offshore (*96*, *97*, *99*, *100*). Nonetheless, highly bioturbated (up to BI = 5) glauconitic sandstone beds with brachiopod, hyolith, and trilobite fragments denote prolonged colonization windows, coincident with transgressive intervals of near-normal marine salinities (*96*). Similar glauconitic siltstone and sandstone with thin shaly interbeds characterize SCF-yielding horizons from the Cambrian stage 4 Colville Hills drillcore locality and early middle Cambrian, sandstone-dominated Mount Clark Formation outcrops, both of the Northwest Territories, Canada (*23*, *101*, *102*). These shallow marine, markedly storm-influenced facies host deposit and suspension feeder burrows produced during fair-weather intervals, and commonly show BIs of 3 to 4 and locally up to 5 (*101*, *102*). Sedimentologically and ichnologically similar shale-sandstone horizons have yielded SCFs in the broadly coeval Mahto Formation (*60*) and the File Haidar Formation of Sweden (*62*).

In this light, the depositional context of the nearshore-offshore transitional BAF, hosting normal marine epifauna as well as unusually high local bioturbation intensities up to BI = 6 (*37*, *39*, *46*), suggest a paleoecological optimum along the continuum of Cambrian BST-macrofossil and SCF localities: a well-aerated, relatively low-energy, upper offshore/lower shoreface subequatorial setting, intermediate between oxygenated but freshwater-influenced/higher-energy nearshore environments on the one hand and dysoxic offshore/marginal settings on the other (Fig. 1A). A dearth of dark shale with amorphous organic matter accumulations (*37*), photosynthetic microorganisms [figs. S3, H to J, and S2, A and B; Supplementary text; (*37*)], and high abundances and densities of animal traces (*37*, *39*, *44*) are all consistent with deposition in a relatively well-oxygenated environment with abundant primary production, sustaining intense and protracted metazoan activity (*37*, *39*, *44*).

## DISCUSSION

The Bright Angel SCF assemblage provides an exceptional preservation window into a middle Cambrian shelf biota. Its components display an unparalleled size range and degree of articulation: The most complete (e.g., Figs. 2A and 3L) exceed the 10^1^- to 10^2^-μm size range of previously recorded Cambrian SCFs (*3*, *48*) by an order of magnitude. Hence, the Bright Angel specimens permit a detailed reconstruction of functional morphologies and their adaptive significance and partly break the inherent trade-off in completeness versus abundance and distribution that has constrained the study of SCFs (*3*). In light of the Grand Canyon’s extensive sedimentological and ichnological records, these exceptional fossils reveal phylogenetically derived and functionally sophisticated Cambrian metazoans inhabiting a comparatively “normal” marine environment: an oxygenated, productive, and extensively bioturbated setting, where the most severe limiting factors affecting other broadly coeval exceptionally preserved faunas, including oxygen scarcity (*4*, *22*), desiccation (*22*), and nutrient availability (*20*), would have been comparatively attenuated or absent.

Against this paleoenvironmentally permissive (*28*) background, the Bright Angel SCFs suggest a relatively escalated ecology by middle Cambrian standards. The first line of evidence for escalation is their comparative taxonomic modernity [(*28*, *30*)]. Emerging evidence indicates that conventional BST-macrofossil biotas hosted ecologically important members of relatively derived major clades, such as mandibulate and chelicerate arthropods (*7*, *103*–*105*), in addition to the stem-group relatives of modern phyla. Even so, most SCF types in the Bright Angel biota differ from such macroscopic counterparts by being attributable with varying degrees of confidence to extant classes. Branchiopod elements indicate a coexistence of at least two closely related crown crustacean taxa, implying a degree of ecological partitioning reflected by their fine-scale differences in molar ornamentation. Meanwhile, aplacophoran-like radulae suggest the presence of molluscs with crown-group affinity (*60*). This predominance of derived taxa among the Bright Angel SCFs fits an important prediction of the escalation hypothesis: permissive settings as cradles of phylogenetic novelty, offering potential starting points for the ecological spillover of derived clades into progressively more marginal environments (*28*, *30*).

As predicted by the escalation hypothesis, the taxonomic modernity of the Bright Angel SCFs is also complemented by derived functional morphologies, which show marked functional coupling, integration, and specialization of preserved feeding structures. By analogy with living counterparts, the multielement particle capture, transport, filtering, and triturating apparatus of the BAF crustaceans implies a degree of anatomical subfunctionalization and active food processing. This feeding mode differs markedly from the metabolically undemanding, semi-sessile passive suspension feeding inferred for early-diverging panarthropods from BST-macrofossil biotas, epitomized by luolishaniid lobopodians (*8*, *59*). The combined thoracic, setal, and molar complex of the BAF crustaceans also contrasts with the relatively undifferentiated and functionally uncoupled appendages of the hymenocarine (*7*) and radiodont (*9*) planktivores of BST-macrofossil faunas. The differentiated scales and incisors of the BAF branchiopods show that by the middle Cambrian, this versatile functional complex had been adapted to diverse particle feeding and potentially predatory roles, coexpressed within the same habitat. Similarly, the chains of robust boot-shaped teeth seen in the Bright Angel radulae are best interpreted as part of a multi-echelon, conveyor belt–like apparatus (*60*), offering a more extensive and specialized scraping girdle than the few, short, and dissimilar rows of comparatively delicate teeth found in BST lophotrochozoans (i.e., *Wiwaxia* and *Odontogriphus*). In keeping with the escalation hypothesis, these derived feeding devices represent new “emergent wholes” with raised functional standards (*28*), breaking the mechanical trade-offs inherent in less integrated or subfunctionalized precursors.

Uniquely among SCF biotas, the unusual morphological complexity of the Bright Angel priapulomorphs also suggests a phylogenetically diffuse “raising of performance standards” that extended to otherwise typical elements of the soft-bottom Cambrian evolutionary fauna. The diverse pharyngeal teeth of *Kraytdraco* point to an internally differentiated but functionally coherent scraping/raking and “filtering” feeding apparatus, partly convergent on modern deposit-feeding counterparts. The morphologies of these sclerites denote an investment in sophisticated fine-scale cuticular architectures that is unmatched among fossil and living priapulids (*50*) and similar to the degree of elaboration expressed by the plumose, branching, or pappose substructures of arthropod setae [compare with Fig. 7 and fig. S10; see (*106*)]. The morphological complexity of the teeth of *Kraytdraco* is most notable when compared to other Cambrian forms for which sclerite morphologies are known down to the micrometer scale: *Ottoia*, *Selkirkia*, *Baltiscalida*, and *Goniomorpha* (*50*, *51*). None of these taxa show tooth projections with recursive branching substructures, and even the distal bristle-like structures of *Ottoia* type A and D teeth consist of short, unspecialized hairy extensions grading into simple spines (*51*). Similarly unbranched fine hairs characterize the teeth of putative detritivorous priapulids from the Kaili biota (*17*). Despite their conspicuously more elaborate cuticular specializations, *Kraytdraco*’s proximal teeth represent a recognizable elaboration of the basic cuspidate geometry found in all macrophagous Cambrian stem priapulids. This morphology is consistent with potential stem priapulid [or even stem-group ecdysozoan (*107*)] affinities (*50*). Their triangular outlines and marked prongs (e.g., Figs. 2, M and R, and 4, C and L) contrast with the transverse pectinate or fimbriated counterparts of extant raking or scraping taxa and the delicate fimbrillae of *Tubiluchus* (see Results). Hence, *Kraytdraco* suggests an independent early origin of priapulomorph microphagy, decoupled from miniaturization and meiofaunal habits (*50*) and exploiting a different morphological pathway than any extant lineage: a sophisticated, if ultimately extinct, feeding adaptation in the most abundant group of Cambrian endobenthic worms (*108*, *109*).

The BAF’s record of phylogenetically disparate feeding innovations is complemented by the conspicuous absence of some otherwise cosmopolitan, ecologically tolerant Cambrian metazoans (*3*, *17*, *22*, *51*, *62*, *79*, *87*, *110*). Among these “pioneer taxa” ranging from dysoxic deeper-water to episodically subaerial settings (*22*) are the carnivorous to detritivorous priapulids *Ottoia* and *Selkirkia*, and the lophotrochozoan grazer and/or detritivore *Wiwaxia*. Because of their tolerance of suboptimal environments, such adaptable generalists tend to dominate resource-limited early successional biotas (*22*) and are prevalent during the early phases of food web assembly (*111*, *112*). The exceptionally wide range of ecophysiological tolerances shown by wiwaxiids, selkirkiids, and ottoiids suggests that their absence from the Bright Angel assemblage is not readily explained by abiotic stressors, particularly given that the BAF’s inner detrital shelf setting falls well within the paleoenvironmental extremes of their distribution. By contrast, the biotic context of the Bright Angel SCF biota is consistent with exclusion of less competitive pioneer taxa (*22*) by later-successional and comparatively specialized forms, relegating them to more physiologically marginal (*28*) settings.

**Fig. 10.**
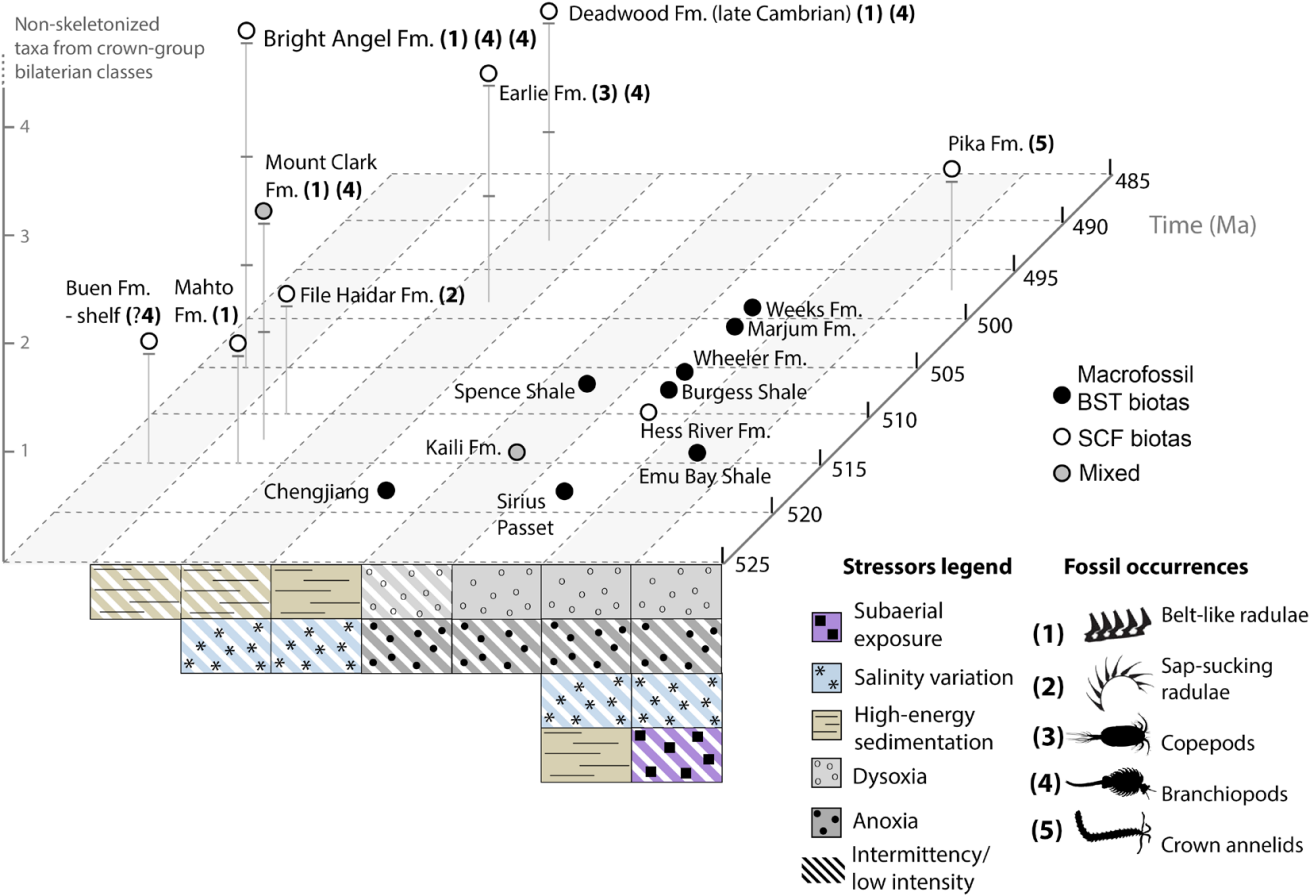
Paleoenvironmental variation and faunal modernity in representative Cambrian organically preserved biotas. Occurrences of non-skeletonized fossils from bilaterian crown-group classes are plotted in relation to time and the abiotic stressors represented in the bar chart at bottom. Associated preservational windows (macro-BST versus SCF biotas), taxonomic occurrences, and color-coded stressors are indicated in the legend to the right. For each stressor in the bar chart, diagonal stripes indicate intermittency and/or low intensity. The order of localities on the horizontal axis reflects, from left to right, a generalized gradient from higher to lower habitability, as reflected by each combination of stressors. Data for the BAF refer to the nearshore-offshore transitional setting hosting the newly described SCF biota, and not to the full paleoenvironmental spectrum of the formation as a whole; likewise, inferred stressors for other plotted biotas refer to the relevant fossil-bearing intervals. Radulae silhouettes reproduced from (*114*) under the terms of the Creative Commons Attribution License http://creativecommons.org/licenses/by/4.0/. Copepod (https://creativecommons.org/publicdomain/zero/1.0/; uploaded by Guillaume Dera), branchiopod (https://creativecommons.org/publicdomain/mark/1.0/; uploaded by Thomas Hegna), and flebelligerid annelid (https://creativecommons.org/publicdomain/zero/1.0/; uploaded by Kanchi Nanjo) silhouettes sourced from phylopic.org. Data after (*10*–*12*, *14*, *17*, *18*, *22*–*26*, *51*, *58*, *60*, *62*, *63*, *79*, *114*, *115*, *118*) and the present study.

This escalatory scenario is consistent with the paleoecological signatures of other SCF localities. Branchiopod suspension feeding apparatuses and substrate-scraping radulae from the Earlie-Deadwood formations, similar to BAF counterparts, are hosted by shales interbedded with conspicuously burrowed (*25*) glauconitic sands in an otherwise scarcely bioturbated shallow marine succession (*96*, *97*, *99*, *100*). These modern forms associated with high bioturbation in epeiric waters suggest a partial analog to the Bright Angel SCF biota: Although they may have been affected by more challenging sedimentation regimes and salinities, stemming from the more marked deltaic influences (*96*, *97*, *99*) and possibly lower salinities of the Western Canada sedimentary basin (*113*), the Deadwood SCF faunas probably lived under relatively habitable and well-aerated conditions (*24*, *96*). The makeup of the Earlie-Deadwood SCF fauna appears correspondingly intermediate between that of the BAF biota and more physiologically marginal (*28*) communities experiencing the more severe stresses of dysoxia and/or periodic emergence (Fig. 10): The Deadwood branchiopod and mollusc SCFs are variously associated with wiwaxiids and ottoiids, sometimes within the same horizons [see (*25*) and table 1]. Similarly mixed assemblages, hosting functionally modern radulae among otherwise typical BST wiwaxiids and priapulids, characterize the shallow marine deposits of the stage 4 Colville Hills (*23*) and File Haidar biotas (*62*, *114*). However, modern-style molluscs and crustaceans are absent in deeper-water, dysoxic early middle Cambrian SCF biotas like the Kaili assemblage (*17*) and in more recent (latest middle Cambrian) peritidal counterparts from the Pika Formation, where members of extant classes are restricted to few hypertolerant benthic generalists (*22*). Notably, an absence of the modern-style taxa of the Bright Angel, Deadwood, and Mount Cap/Clark faunas from the offshore, dysoxic setting of the early to middle Cambrian Hess River biota, situated at a similar paleolatitude and also expressing submicrometer-scale preservation of delicate and semi-articulated SCFs, argues against first-order latitudinal, biostratinomic, or taphonomic controls on these faunal discrepancies [see (*115*)]. This emerging record of overlapping taphonomic modes (SCF and macro-BST) across a broad gradient of Cambrian paleoenvironments also challenges postmortem transport as an explanation for these observed faunal differences: The potential for both macro-BST and SCF carcasses to undergo substantial transport does not explain why less phylogenetically or functionally modern forms should be systematically displaced to less habitable depositional settings, regardless of carcass size or composition (*115*). Moreover, remarkably articulated material from the BAF (e.g., Fig. 2A) suggests comparatively limited reworking of carcass fragments.

The fit between these paleoecological patterns and the escalation hypothesis suggests a preliminary model for the distribution of Cambrian SCF and BST-macrofossil faunas: one in which both recency and habitability are predictors of functional and phylogenetic modernity (Fig. 10). The permissive habitat, lack of early-successional pioneers, and co-occurring behavioral proxies of the Bright Angel SCF fauna suggest an upper end member of the habitability spectrum of mid-Cambrian biotas. Locally, a correspondingly widespread elevation of biotic performance standards (*28*) characterizes both taxa from extant classes and otherwise typical members of the Cambrian evolutionary fauna. Cambrian epicratonic faunas that may have experienced somewhat higher levels of abiotic stress, and/or from earlier time intervals (Fig. 10), tend to include modern-style groups together with tolerant BST generalists. Toward the opposite paleoecological extreme are Cambrian biotas affected by severe limiting factors on metabolic and functional performances, above all dysoxia: the physiologically marginal “refugia” of the escalation hypothesis. Their ecologies are dominated by relatively archaic BST forms (*3*) and invaded by ecophysiologically tolerant members of extant classes only in their youngest known Cambrian examples, such as the Pika biota (Fig. 10).

These laterally translated patterns of faunal distribution through time (Fig. 10) suggest a protracted spillover of increasingly derived forms from permissive into more stressed or metabolically limiting environments, as predicted by the escalation hypothesis. This model is based largely on the stage 4 and middle Cambrian deposits that have yielded the most SCF and BST-macrofossil biotas (Fig. 10). However, chronologically older faunas match its predictions. Although it remains less comprehensively characterized than other BST biotas (*13*), the dysoxic, deeper-water stage 3 Sirius Passet Lagerstätte may have hosted a relatively archaic “Terreneuvian-style” (*116*) ecology dominated by early-diverging gilled lobopodians, non-biomineralized macroarthropods, and chaetognath apex predators (*13*, *116*). Among broadly coeval, stage 3 biotas, well-aerated shelf settings from the Buen Formation have yielded recognizable crustaceomorphs (*26*). Meanwhile, comparatively oxic but moderately stressed environments of a similar age, prone to freshwater discharges and high-energy deposition as in the case of the Chengjiang biota (*11*), hosted classic BST-macrofossil faunas similar to those of younger (Miaolingian) deeper-water deposits (*4*, *5*, *11*, *14*). These patterns of faunal distribution fit both proposed Phanerozoic-wide trends of shallow marine origination and later offshore displacement in major biomineralized groups (*117*) and independent evidence for escalation as a driver of Cambrian macroevolution, as manifested in predator-prey arms races (*34*) and the upward “ratcheting” of the physiological costs and functional efficacy of early animal skeletons (*32*).

By coupling extensive ichnological and sedimentological records with exceptional body fossils, the BAF points to an unexpectedly escalated ecological baseline for Cambrian communities beyond variously marginal habitats: one that foreshadows the increasing taxonomic and functional modernity of later Paleozoic faunas. The ratcheting of adaptive innovations in such “Goldilocks zones” of Cambrian habitability, left largely unrecorded by conventional BST-macrofossil deposits, may have imposed a lasting directional imprint on the trajectory of Phanerozoic macroevolution. Open-ended escalatory feedbacks in resource-rich settings would have locked in the incentives for the assembly of modern functional strategies, regardless of which clades ultimately proved most successful in enacting them. The ensuing spillover of increasingly complex, integrated, and versatile phenotypes offers a potential explanation for the gradual marginalization of Burgess Shale–style ecologies. Moreover, it hints at long-term competitive sorting as an important determinant in the establishment of phylogenetically modern actors. If the Cambrian Explosion laid the foundations of modern metazoan adaptive solutions (*1*), it is the scaling up of their competitive interactions that may have enforced directional, long-term trends of functional innovation in the Phanerozoic biosphere.

## MATERIALS AND METHODS

We collected a total of 29 samples of diverse shale lithologies, ranging from massive to fissile, gray to purple, and with various degrees of weathering and dolomitization, along an east-west transect of the BAF in the Grand Canyon National Park. Two of the sampled localities, yielding finely laminated, fissile, and moderately micaceous green-gray shale from the lower to middle BAF in the central, normal marine facies of the Grand Canyon [Fig. 1, C and D; see (*35*, *40*)], were productive (data S1). We extracted SCFs following the low-manipulation procedure detailed in (*3*). Shale samples were dissolved by gentle maceration in 40% concentrated hydrofluoric acid. We filtered the resulting residue (max. ~300 g per sample) through a sieve with 60-μm mesh size. SCFs were handpicked from the residue under a Zeiss Stemi SV 11 binocular stereomicroscope using a pipette, left to decant in deionized water, and mounted on glass coverslips for scanning electron microscopy (SEM) and optical microscopy. Representative specimens were photographed under a Zeiss Axioplan 2 stereomicroscope equipped with a Kontron Elektronik ProgRes 3012 camera. SEM visualization was performed in the Department of Earth Sciences, University of Cambridge using a Quanta-650F field-emission gun scanning electron microscope.

## Supplementary Materials

### The PDF file includes:

Supplementary Text Figs. S1 to S11 Legend for data S1 Legend for movie S1 References
