# Supplementary material for: Evolutionary escalation in an exceptionally preserved Cambrian biota from the Grand Canyon (Arizona, USA)
Source: Sci Adv. 2025 Jul 23;11(30):eadv6383. doi: 10.1126/sciadv.adv6383 (PMC12285723; doi:10.1126/sciadv.adv6383)
Supplement: Supplementary file 1 — Supplementary Text Figs. S1 to S11 Legend for data S1 Legend for movie S1 References [file sciadv.adv6383_sm.pdf]

Science Advances
AAAS

Supplementary Materials for

Evolutionary escalation in an exceptionally preserved Cambrian biota from the Grand Canyon (Arizona, USA)

Giovanni Mussini *et al.*

Corresponding author: Giovanni Mussini, gm726@cam.ac.uk

Sci. Adv. **11**, eadv6383 (2025)
DOI: 10.1126/sciadv.adv6383

The PDF file includes:

Supplementary Text
Figs. S1 to S11
Legend for data S1
Legend for movie S1
References

Other Supplementary Material for this manuscript includes the following:

Data S1
Movie S1

Supplementary Text

1. *Systematic Palaeontology*

Superphylum ECDYSOZOA Aguinaldo et al. 1997 SCALIDOPHORA Total group of

Phylum PRIAPULIDA Delage & Hérouard, 1897 Incertae familiae

Genus KRAYTDRACO nov.

Etymology

From the Latin *draco* (dragon). After the worm-like, toothed, and canyon-dwelling "Krayt dragon" of the Star Wars fictional universe, in reference to the morphology, comparatively impressive size, and geographical provenance of the fossils.

Type species

Kraytdraco spectatus sp. nov. by monotypy.

Diagnosis

As for type species.

Kraytdraco spectatus sp. nov. Figs. 2-4, figs. S4-S8

Etymology

From the Latin *spectatus* (remarkable, illustrious) referring to the exquisite preservation and size of the carbonaceous fossils attributed to this taxon.

Holotype

Fig. 2A; slide GM23-140L-10, England Finder coordinates: p37. Designated paratypes: Figs. 2-4, figs. S4-S8. England-finder coordinates provided in image captions.

Referred material:

GM23-140L-1 to -42; GM23-140L-SEM-1 to -2 (data S1).

Diagnosis

Priapulid-grade scalidophoran with a bipartite pharynx, comprising two tracts of different width separated by a tapering transitional zone and surrounded by polygonally patterned cuticle when inverted. Teeth in the wide (distal) tract of the everted pharynx tongue-like, with U- to V-shaped arch, and with coniform spine-like denticles interspersed with elongated unbranched filaments. Teeth in the transitional zone between the two tracts forming a continuum of U- to V-shaped elements with dendritic denticles, terminating apically into a multi-cuspidate prong, and showing prominent transverse rows of comb-like projections on their median surface. Teeth in the narrow (proximal) tract triangular, equilateral to isosceles, with falcate profile closest to the base of the eversible pharynx, and with prong multicuspidate in distalmost specimens and unicuspidate in proximal-most specimens. The arches of teeth in the narrow tract bear denticles that grade from dendritic, finely splintering filaments to unbranched filaments with club-like terminations.

Distribution

Middle shale members of the middle Cambrian Bright Angel Formation, Grand Canyon National Park, Arizona, USA.

2. *Palaeontological context: Ichnofossil record of the Bright Angel Formation*

The BAF hosts a diverse and abundant ichnofossil record. The BAF's trace fossils span at least 21 ichnogenera tentatively attributed to panarthropod, molluscan, annelid, and priapulid makers (*37, 39, 46, 120, 121*). This record is dominated by vermiform traces, which comprise 77% of the BAF's recorded ichnotaxa (*39*).

In the Grand Canyon, the BAF's trace fossils are distributed across two intergrading but recognisably distinct ichnofacies: the *Skolithos* and *Cruziana* ichnofacies (Fig. 9F; (*37, 39, 46*). The BAF's *Skolithos* ichnofacies is typically hosted by well-sorted, cross-bedded to massive sandstones deposited under wave or current-influenced conditions, denoting probable foreshore to middle shoreface deposits (*36, 37, 39, 47*). It is distinguished by a low disparity of trace fossils, dominated by full-relief vertical burrows produced by probable suspension feeders: the U-shaped *Arenicolites* and *Diplocraterion* (Fig. 9D) and the subcylindrical *Skolithos.* These burrows co-occur with sparser shallow locomotory trails, such as *Aulichnites* and *Scolicia* (*37, 46, 120*).

The BAF's *Cruziana* ichnofacies corresponds to a broader distribution from lower shoreface to lower offshore, and is more representative of the fully marine facies of the central Grand Canyon where the new Bright Angel SCF biota occurs (Fig. 1C-D; (*36, 39, 47*)). This ichnofacies comprises morphologically disparate, shale and sandstone-hosted full-relief and semi-relief fossils (Fig. 9F). Its most common ichnotaxa are the spreiten-bearing tubular burrow *Teichichnus* and the passively filled, subcylindrical, horizontal and thinly lined *Palaeophycus* (Fig. 9A-B). Together, these putative domichnia (dwelling traces) produced by vermiform bilaterians span the full palaeo-bathymetric range of the *Cruziana* ichnofacies (*39, 46*). Other, less prevalent fodinichnia (i.e., feeding traces) produced by wormlike organisms include subhorizontal semi-cylindrical traces, such as the passively infilled *Fucusopsis* and the actively infilled *Planolites*. Among non-vermiform traces, arthropod scratch marks (Fig. 9E) reflecting substrate scraping or grazing (e.g., *Monomorphichnus, Dimorphichnus*) are the most common. Arthropod trackways (e.g., *Angulichnus*, *Cruziana, Diplichnites*) and resting traces (*Rusophycus*) frequently occur in the same facies ((*39*), fig. 17). Other, less common traces include shallow, millimetre-scale ridged trails produced by possible gastropod molluscs (*Cochlichnus*), rope-like sediment 'probes' likely left by priapulids (*Treptichnus*), plug-like impressions made by putative anthozoans (*Bergaueria*), and rare, rounded paired imprints (*Bicavichnites*) possibly produced by walking lobopodians (Fig. 9F (*37, 39, 46, 120, 121*)).

3. *Non-metazoan elements and unattributed cuticles*

Figs. S2-3; data S1.

Description and remarks

Non-metazoan fossils with recalcitrant extracellular walls have been documented together with similarly acid-resistant animal counterparts in virtually all known SCF assemblages (*3, 17, 50*). These organic remains encompass diverse microbial problematica, ranging from microalgae to putative fungi and bacterial assemblages to spheroidal 'acritarchs' of uncertain affinities. In the BAF, organic filaments and sphaeroidal forms are represented in both eastern and central exposures yielding SCFs or conventional palynomorphs (Fig. 1C; cf. (*37*)).

In the Bright Angel biota, acritarchs are represented by organic-walled spheroids ranging between ~4 to 300 µm in diameter (N=103; figs. S3A-G, S9J, S2F, Figs. 6H-K, 7C). Their outer surface is unornamented, but typically shows a 'mottled' appearance or fine cracks suggesting a thin and relatively delicate wall. Smaller (~4-16 µm wide), translucent subspherical acritarchs lacking surface cracks are found attached to the sternal elements and setal arrays of the Bright Angel crustaceomorphs (Figs. 6H, K, 7C, fig. S9J). Based on their shape, size, lack of ornamentation, and recurrent pattern of surface folding the Bright Angel specimens are identifiable as *Leiosphaeridia*-type acritarchs, a common and environmentally widespread occurrence in Cambrian SCF biotas (e.g. (*22*)). As in other Cambrian metazoan-rich SCF biotas (*3, 17*), acanthomorphic acritarchs are not found in the Bright Angel assemblage.

In addition to *Leiosphaeridia*-type specimens, the Bright Angel 'acritarchs' include a single cuticular fragment showing a pronounced reticulate pattern on its surface (fig. S3N). The reticulate pattern consists of a mesh of polygonal to elliptical units defined by the intersection of biconcave flanges, each approximately 15 to 30 µm across, and with optically dense margins. Each unit in this mesh delimits a web of finer, ~2µm-wide polygons with lighter and less robust walls (fig. S3O). These features find potential counterparts in *Nematothallus*-type cuticles, traditionally attributed to embryophytes but reinterpreted as produced by red algae (*122*). However, the specimen is considered too fragmentary to support an attribution to this genus.

The Bright Angel non-metazoan SCFs also include interwoven filamentous aggregates (N = 10), forming dense mats or tangles up to about 300 µm across (figs. S3H-J, S2A-B). Each aggregate comprises filaments of similar width and morphology, always consisting of non-tapering, strap-like elements. By comparison with known counterparts from across other SCF biotas, these fossils can be identified as of probable cyanobacterial origin, suggesting a photosynthetic habit. This corroborates a depositional environment within the photic zone (*85*) for the Bright Angel biota (*36*). Given the relatively large sieve mesh size (60 µm) used in the present study, these microbial SCFs probably include only a subsample of their original diversity present in the Bright Angel biota.

In addition to likely non-metazoan fossils, the Bright Angel SCF problematica comprise thin, ~200-300 µm -wide cuticular sheets covered by irregularly distributed knob-like protrusions and otherwise lacking diagnostic features (Fig. S3K-M). These cuticles resemble pock-marked arthropodan counterparts from the early Cambrian File Haidar Formation of Baltica ((*123*), fig. 2AC) in their delicate construction and surface ornamentation. However, the closest match is provided by the similarly tuberculate cuticles of the bivalved bradoriid arthropods, previously reported as SCFs from the early Cambrian Buen Formation of Greenland (cf. (*26*), fig. 3; (*124*), fig. 3, 4a). This morphological correspondence may corroborate a possible presence of bradoriids in the Bright Angel biota (cf. (*49*)). However, pending the discovery of more complete material, these SCFs are not conclusively attributed to any taxonomic group.

1. Provenance

Collection of shales was done by our collaborative research team on a Colorado River trip in September 2023 run by K.E.K., with sampling guidance by G.M. Fossils were validated for authenticity by G.M.: authenticity was validated by direct extraction from host shales and by optical and scanning electron microscopy visualization, which confirmed their preservation typical of Cambrian small carbonaceous fossils and showcased morphologies consistent with other coeval taxa and modern counterparts. The fossils were put into stratigraphic and age context by the collaborative team of coauthors. At the time of publication the fossils can be freely accessed at the University of Cambridge, Department of Earth Sciences (Cambridge, UK) during continued research, and will be sent to the Grand Canyon Museum Collection, Grand Canyon National Park (Arizona, USA) for permanent storage.

Supplementary figures

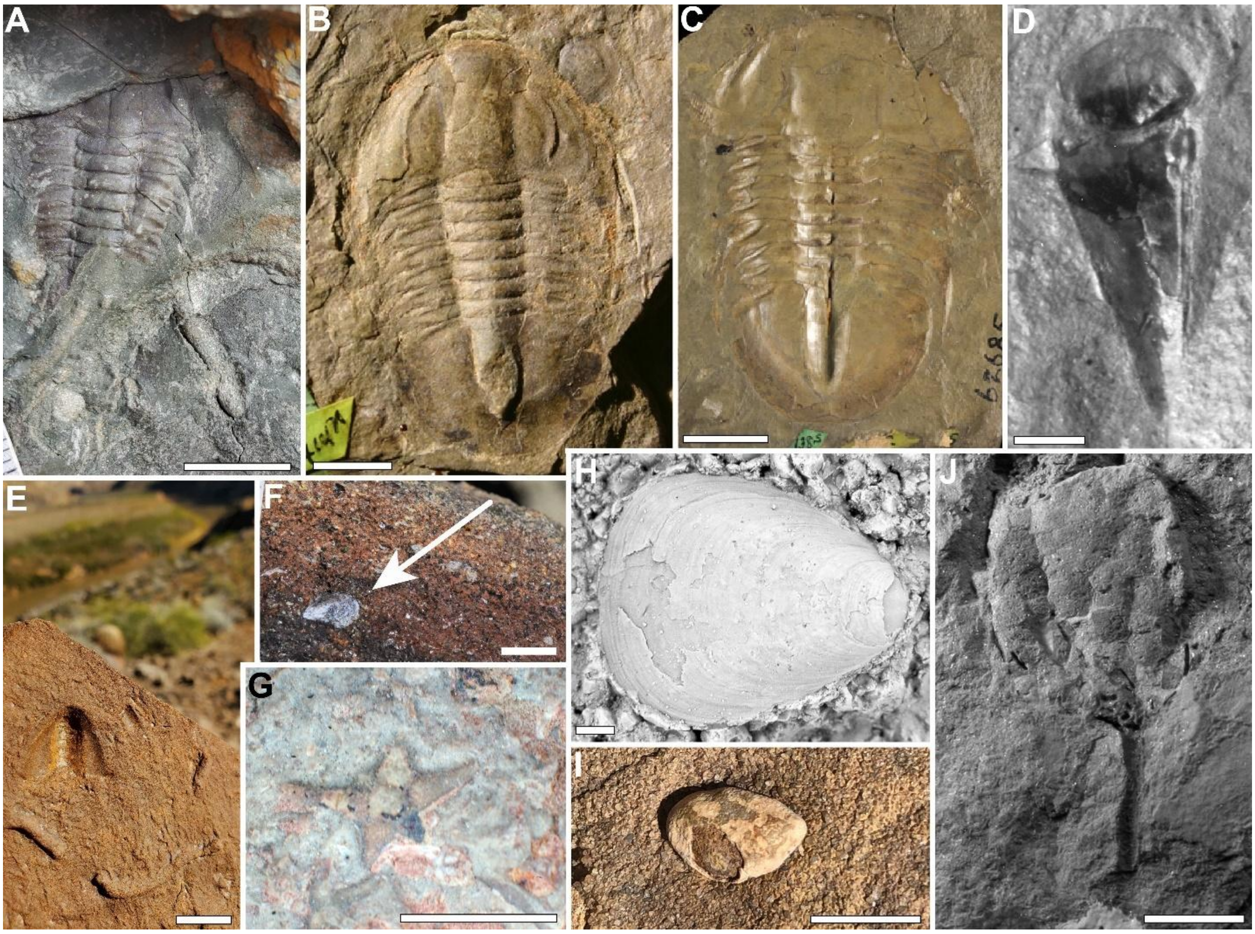

Fig. S1.
Representative biomineralised fossils from the Bright Angel Formation. (A) Trilobite (*Albertella* sp.). (B) Trilobite (*Glossopleura mckeei*). (C) Trilobite (*Anoria tontoensis*). (D) Hyolith preserving conical conch and rounded operculum (grayscale image) (E). *Glossopleura* sp. head fragment with co-occurring tubular trace fossil (bottom) (F) Lingulid branchiopod (white arrow). (G) Pentaradial chanchelloriid spicule. (H) *Lingulella lineata* (FS21-58-6) from Malgosa Canyon. (I) Brachiopod shell fragment. (J) Eocrinoid (*Gogia* sp.); grayscale image. Scale bars: 5 mm except for A-B, J (10 mm) and H (1 mm); scale bars in F and H approximate.

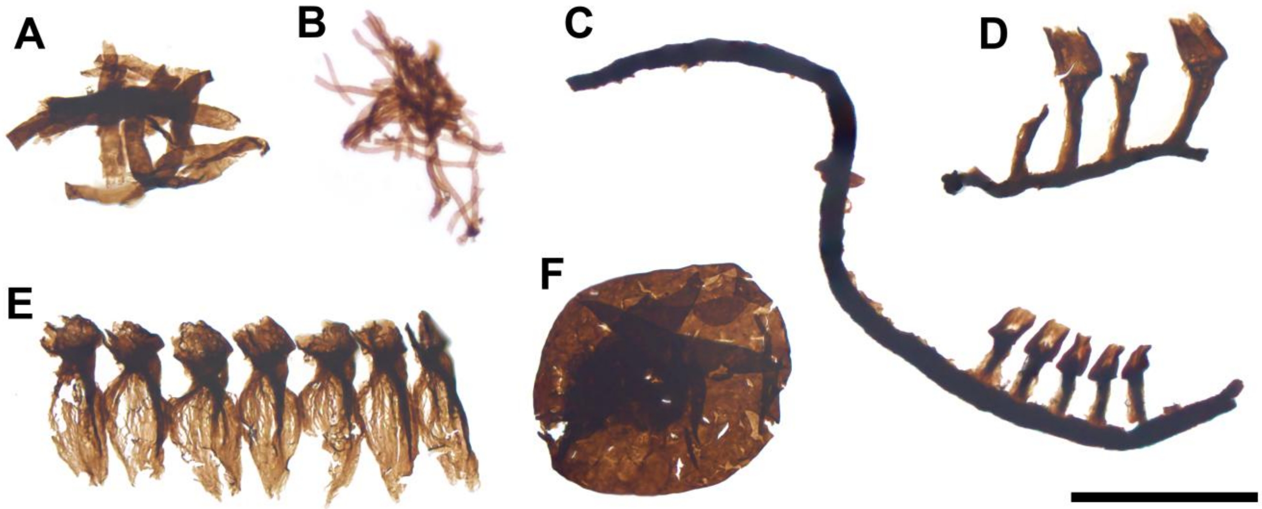

Fig. S2.
Representative organic fossils from the eastern BAF (Red Canyon locality). (A-B) Putative cyanobacterial filaments. (C-E) Radular fragments showing partly articulated fibrous teeth. (F) Sphaeromorphic acritarch. Slide numbers and England Finder coordinates listed in data S1. Scale bar: 100 μm.

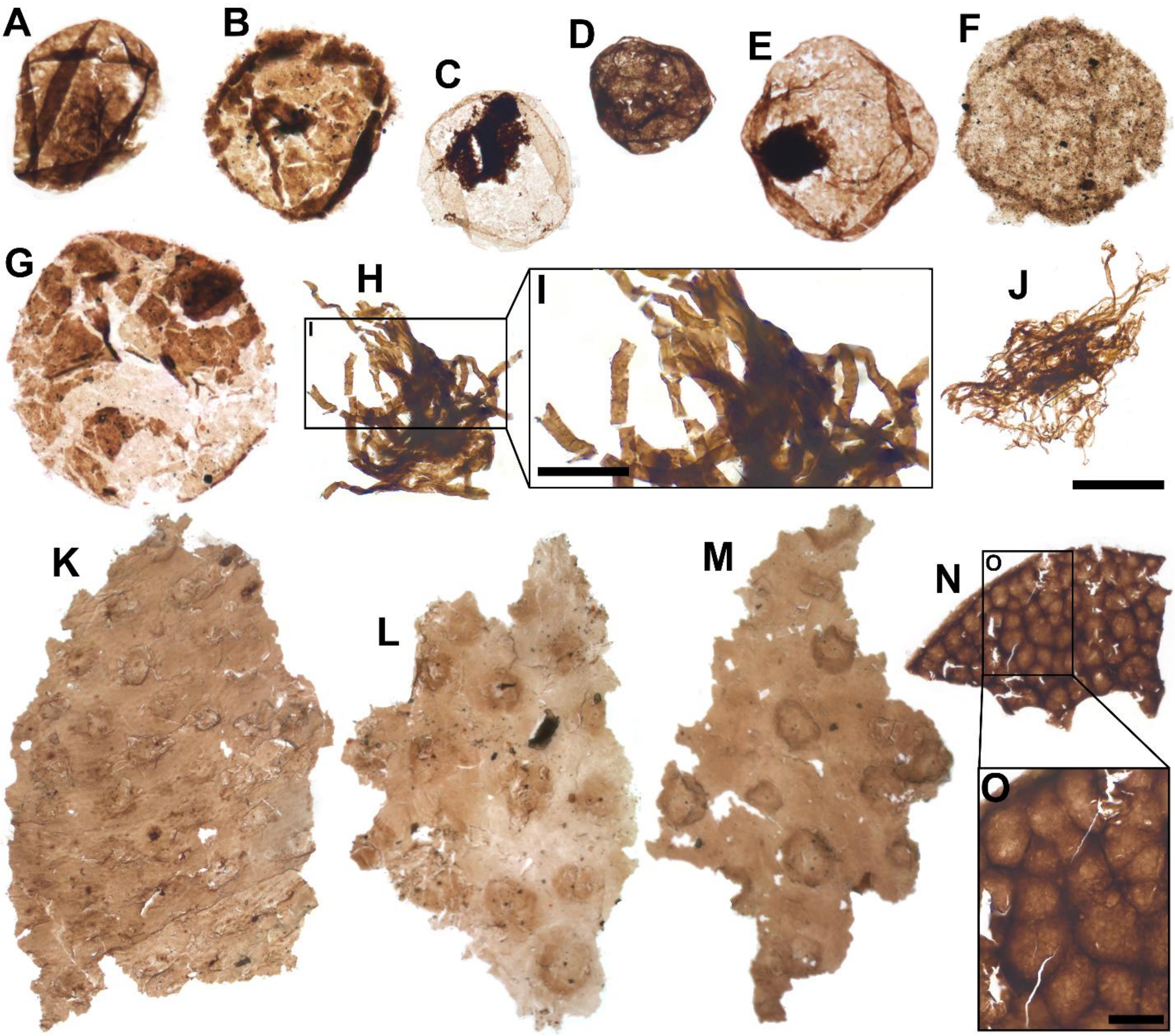

Fig. S3.

Microbial SCFs and cuticular fragments from the Bright Angel Biota. (A-G) leiosphaerid acritarchs showing variable degrees of surface cracking and preservation. (H, J) Filamentous aggregates recording possible cyanobacterial mats. (I) Detail of boxed area in H. (K-M) Tuberculate cuticles from possible bradoriid producers. (N) Possible *Nematothallus*-like cuticle. (O) Detail of boxed area in N showing polygonal construction of the cuticle. Slide numbers and England Finder coordinates listed in data S1. Scale bars: 100 µm except in I (50 µm), O (25 µm).

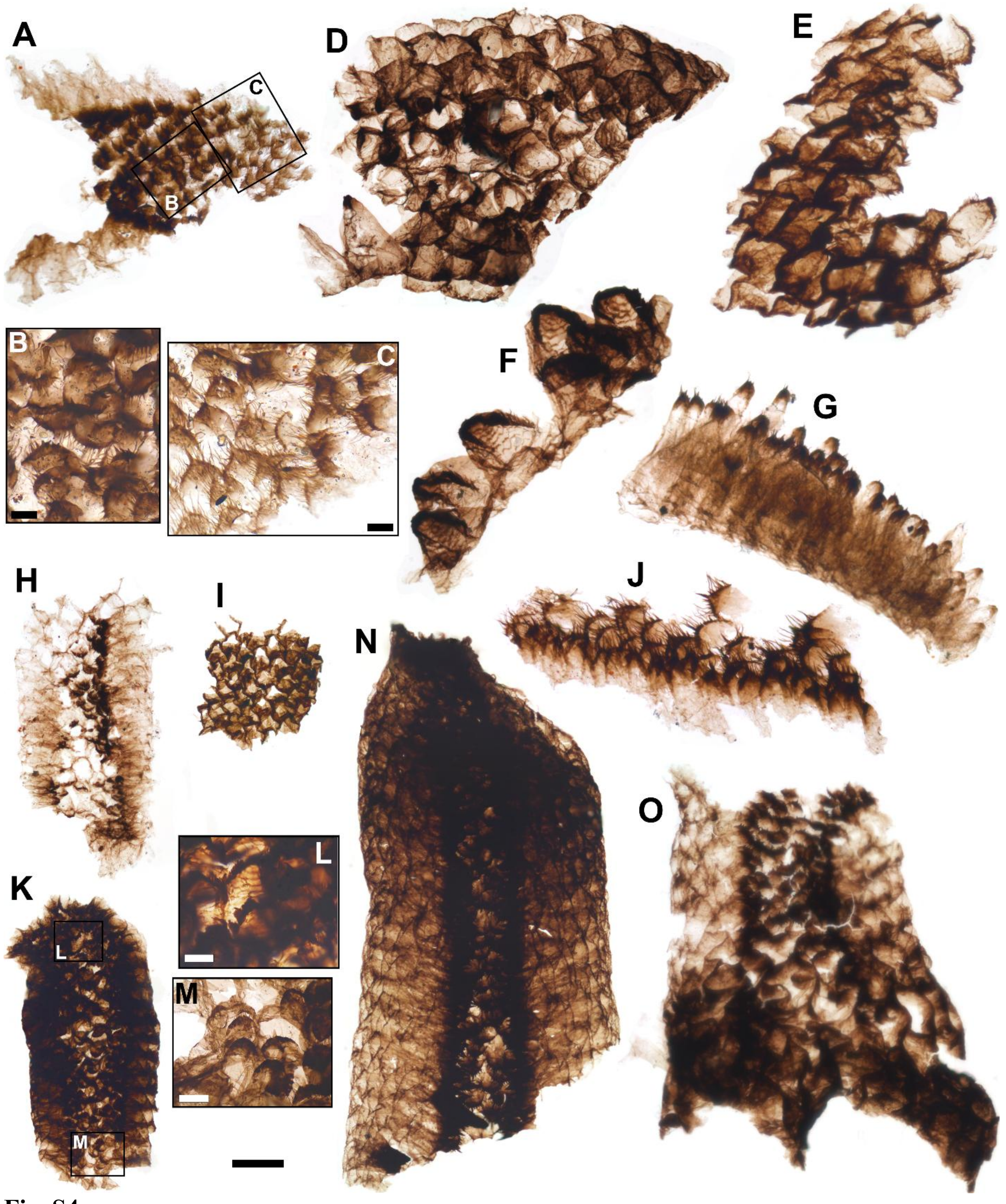

Fig. S4.

***Kraytdraco spectatus* gen. et sp. nov., articulated teeth from the pharyngeal wide tract and transitional zone.** (A) Partly articulated distal tract. (B) U-shaped teeth combining spinose and unbranched filamentous denticles. (C) Slightly more distal teeth showing a more triangular arch. (D-E) Articulated clusters of transitional zone teeth. (F) U-shaped teeth from the distal part of the transitional zone. (G) Articulated cluster of teeth from the proximal region of the wide tract. (H) Section of the wide tract with partly preserved polygonally patterned cuticle. (I) Isolated section of polygonally patterned cuticle. (J) Distal teeth from the wide tract, showing interspersed spine-like and unbranched filamentous denticles. (K) Articulated section of pharynx, comprising part of the transitional zone with teeth bearing comb-like striations and part of the wide tract bearing teeth with interspersed spinose and filamentous denticles. (L) Detail of boxed area in K (rotated) showing

teeth bearing comb-like striations. (M) Detail of boxed area in K (rotated) showing teeth with spinose and interspersed filamentous denticles. (N-O) Articulated sections of the wide tract surrounded by polygonally patterned cuticle. Slide numbers and England Finder coordinates listed in data S1. Scale bars: 50 µm except in B-C (10 µm) H, K, I, N, O (100 µm) and L-M (20 µm).

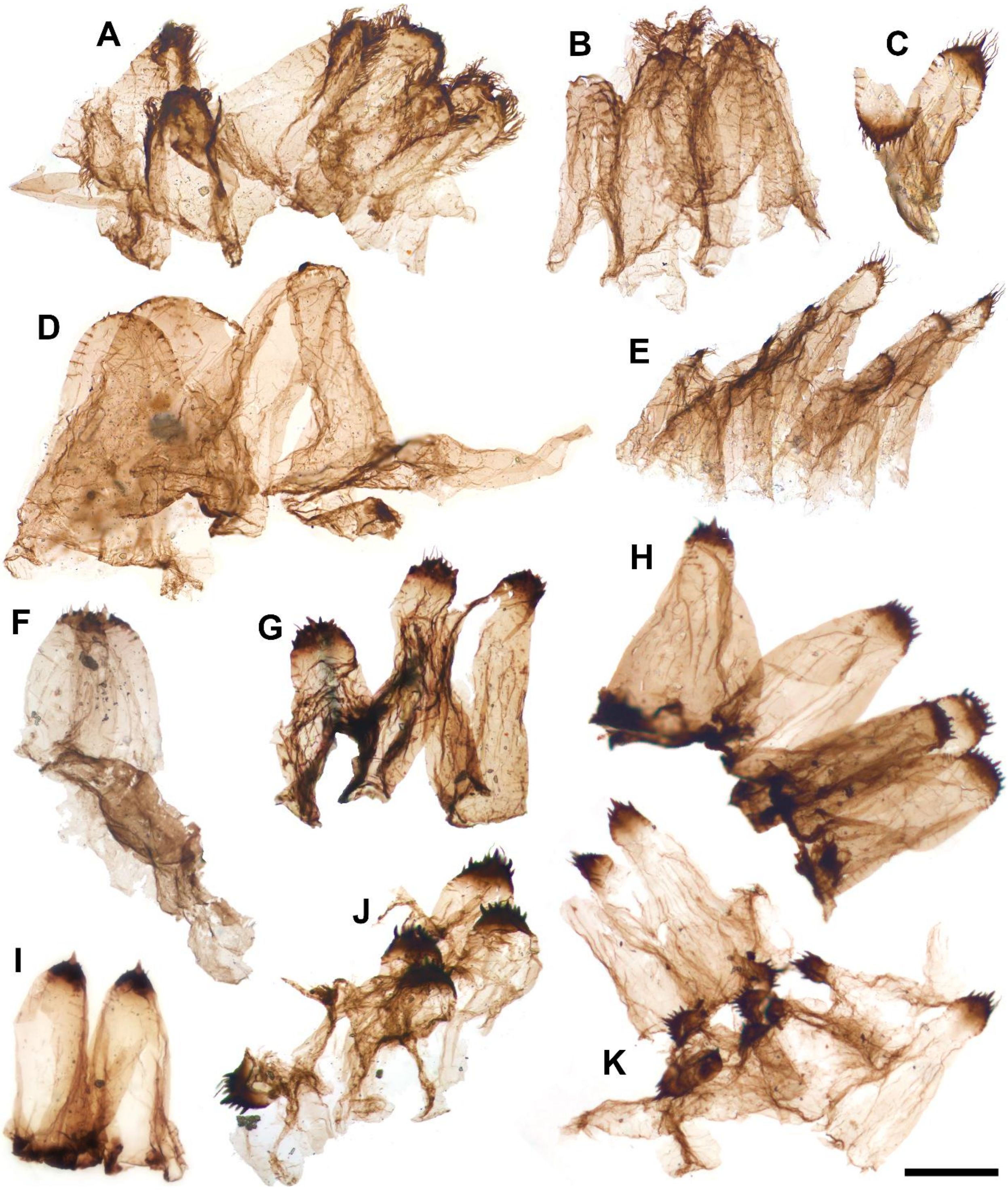

Fig. S5.
***Kraytdraco spectatus* gen. et sp. nov., teeth occupying the pharyngeal transitional zone and wide tract.** (A) Subtriangular (proximal) to U-shaped (more distal) transitional teeth. (B) U-shaped transitional teeth. (C) Wide tract tooth bearing conspicuous comb-like striations and elongated filamentous denticles. (D) Proximal-most teeth of the wide tract (cf. Fig. 4I, right). (E) Wide tract teeth bearing lateral comb-like striations and elongated filaments. (F) U-shaped proximal wide tract tooth. (G-K) Semi-articulated wide tract teeth showing tongue-like delicate basal pads, interspersed unbranched filamentous and spine-like denticles of varying length. Slide numbers and England Finder coordinates listed in data S1. Scale bar: 50 μm.

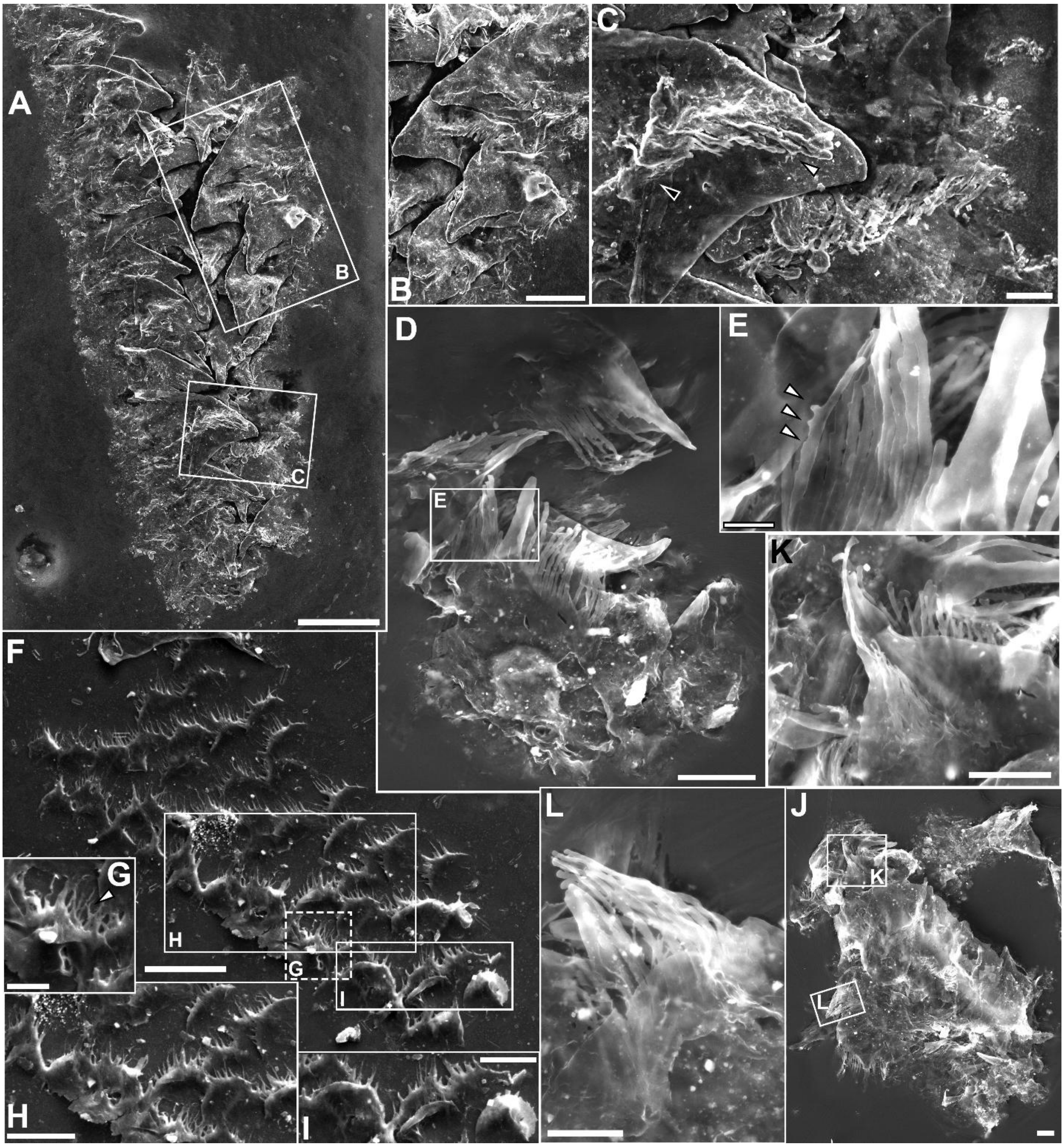

Fig. S6.
***Kraytdraco spectatus* gen. et sp. nov., pharyngeal teeth under scanning electron microscopy imaging.** (A) Section of proximal narrow tract. (B-C) Details of boxed areas in A showing falcate lateral profile of proximal teeth and club-like (white arrowhead) to densely filamentous (black arrowhead) terminations of denticles. (D) Cluster of teeth from the intermediate narrow tract. (E) Detail of boxed area in D showing tubercle-like projections of apical denticles. (F) Series of teeth from the wide tract. (G) Detail of boxed area in F showing tooth with interspersed filamentous and spine-like denticles. (H) Detail of boxed area in F showing transition towards increasingly U-shaped teeth in progressively more proximal regions of the wide tract. (I) Detail of boxed area in F showing U-shaped teeth. (J) Cluster of superimposed proximal tract teeth; details of filamentous outgrowths shown in (K, L). Slide numbers and England Finder coordinates listed in data S1. Scale bars: A, J, 100 µm; B, D, L, 50 µm; C, I, 20 µm; E, G, 10 µm; F, 40 µm; H, K, 30 µm.

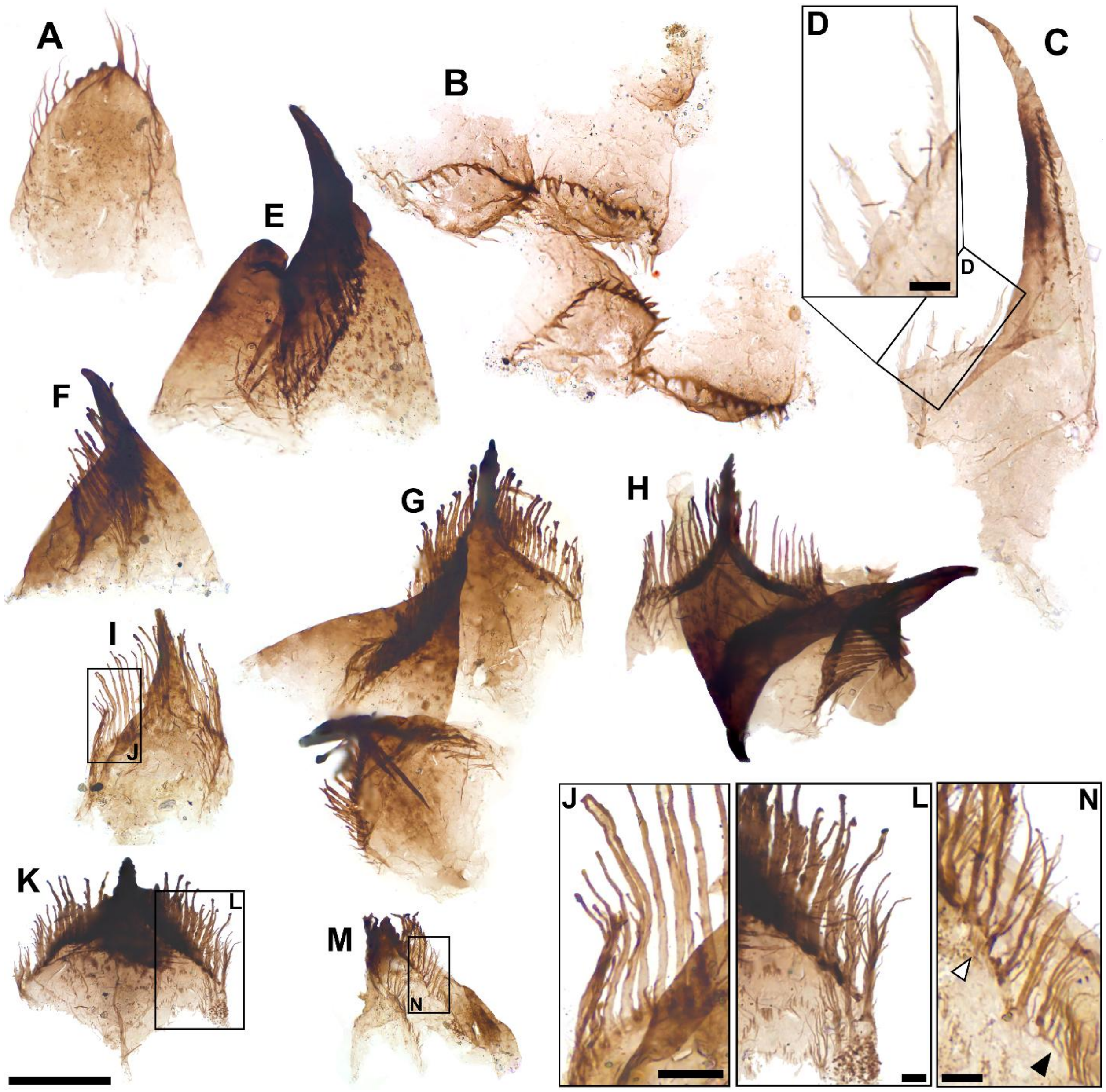

Fig. S7.
***Kraytdraco spectatus* gen. et sp. nov., teeth from the narrow pharyngeal tract and pre-oral scalids.** (A-B), referred elements representing possible denticulate scalids from the trunk region. (C) Introvert hook showing parallel longitudinal rows or ridges of short setulose denticles and basal denticles with side-branching elements. (D) Detail of boxed area in C showing lateral branching of basal denticles. (E) Shorter introvert hook with associated coniform scalid. (F) Possible transitional element between introvert hooks and proximal-most teeth of the narrow tract, showing club-like terminations of denticles. (G) Proximal teeth from the narrow tract in profile, oblique, and frontal views, showing basally restricted branching denticles and apical denticles with club-like terminations. (H) Tooth from the intermediate narrow tract bearing multicuspidate prong, basally restricted branching denticles and apical denticles with tubercle-like lateral projections. (I) Isolated tooth from the narrow tract showing basally restricted branching denticles and apical denticles with club-like terminations. (J) Detail of boxed area in I, showing laterally branching (basal) to unbranched (apical) denticles with club-like terminations. (K) Tooth bridging between intermediate and distal narrow tract elements, combining multi-cuspidate prong, broadly equilateral profile, filamentous denticles with prominent side-branches basally, and apical denticles bearing tubercle-like to short branching projections. (L) Detail of boxed area in K showing denticles with varying basal to apical extent of lateral branching. (M) Distal narrow tract tooth. (N) Detail of boxed area in

M showing basally splintered denticles basally (black arrowhead) and denticles with lateral side-branches more apically (white arrowhead). Slide numbers and England Finder coordinates listed in data S1. Scale bars: 50 μm except in D, J (10 μm), L, N (5 μm).

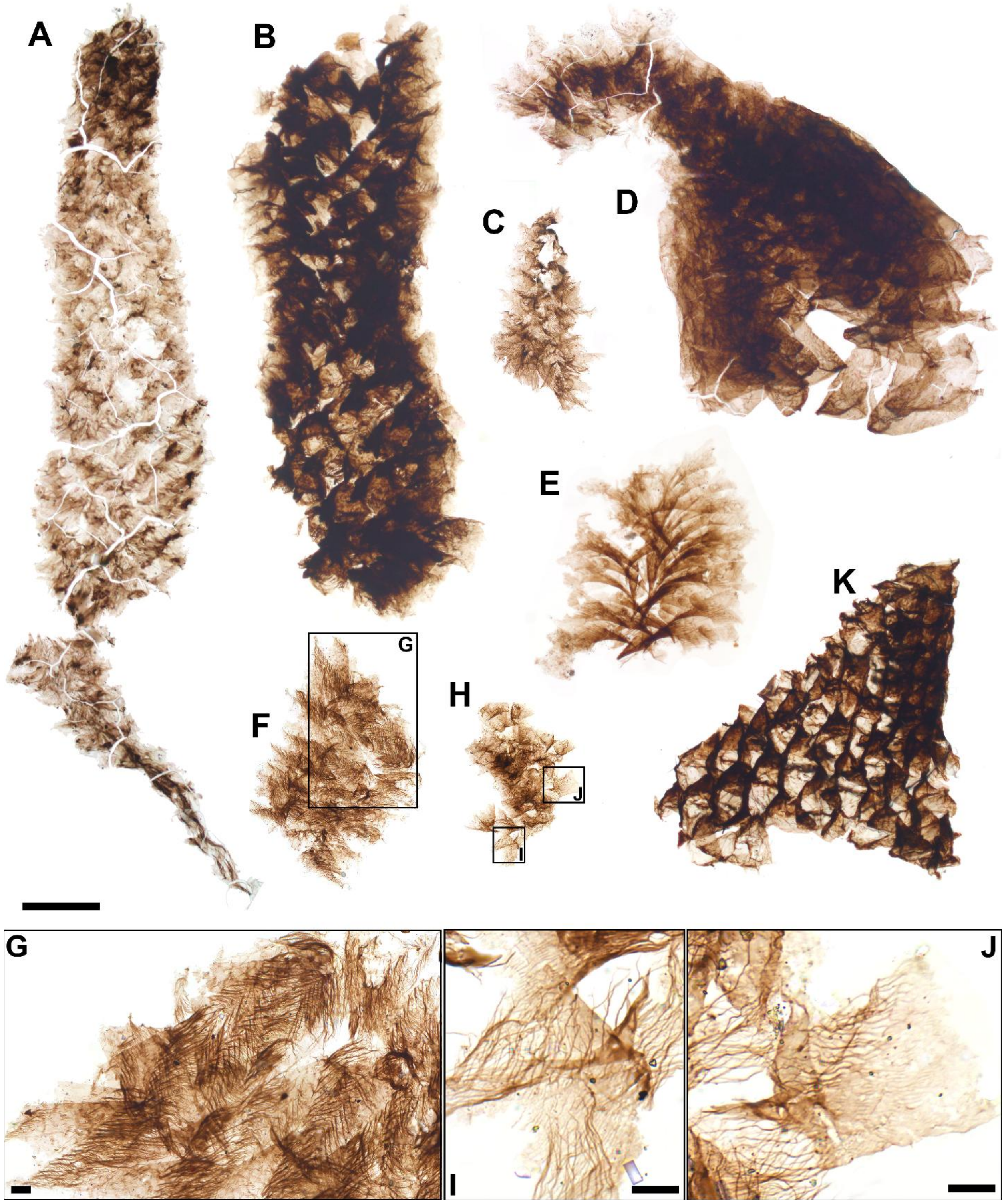

Fig. S8.
***Kraytdraco spectatus* gen. et sp. nov., partly articulated sections of the narrow tract and transitional zone of the pharynx.** (A) Partly preserved narrow tract with delicate cuticle, suggesting a possible moult. (B) Partly preserved narrow tract with robust cuticle. (C) Small specimen recording part of the narrow tract. (D) Partly preserved distal narrow tract grading into transitional zone. (E) Proximal teeth from the narrow tract. (F) Distal part of the narrow tract. (G) Detail of boxed area in F showing teeth bearing filamentous branching denticles and transverse striations of comb-like rows. (H) Portion of intermediate-distal tract. (I-J) Details of boxed areas in H showing teeth with multicuspidate prong and delicate filamentous denticles. (K) Articulated

section of the transitional zone. Slide numbers and England Finder coordinates listed in data S1. Scale bars: 100 µm except in G, I, J (10 µm).

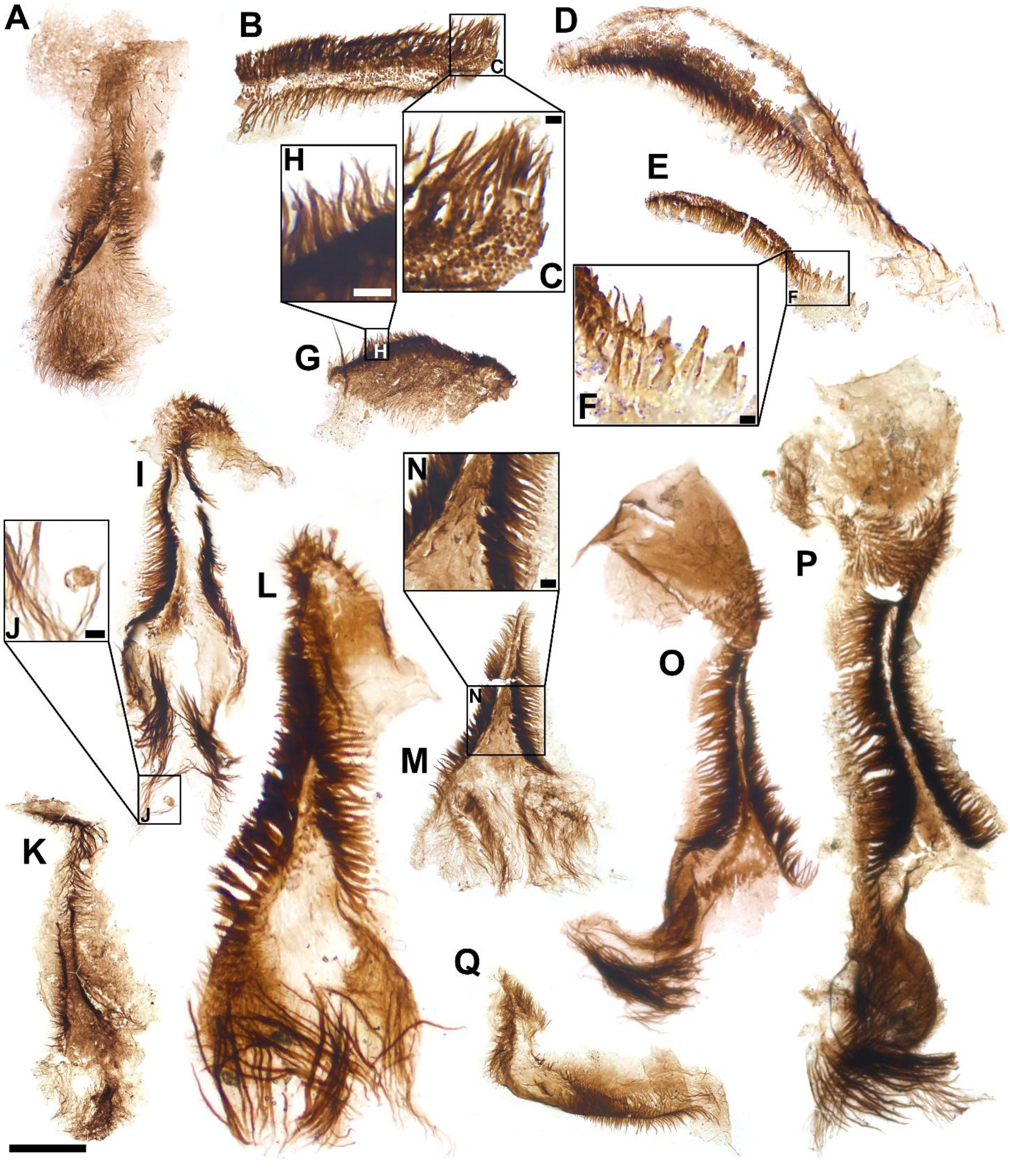

Fig. S9.
Crustacean molars and sternal elements from the Bright Angel Biota. (A, K) Cuticular elements with median strip flanked by setal rows and posterior setulose surface, recording possible invaginated sternal groove elements (cf. (*78*), pl. 20 fig. 8). (B, D-E) Putative Morphotype A molars. (C) Detail of boxed area in B showing setal fringe with lateral setules and molar scales. (F) Detail of boxed area in E showing posterior incisor process part with spines of varying size. (G) Putative Morphotype B molar (left). (H) Detail of boxed area in B showing setal fringe with lateral setules. (I, L-M, O-P) Paragnath-bearing cuticular triangles. (J) Detail of boxed area in I showing sphaeromorphic acritarch medial to the paragnath setae. (N) Detail of boxed area in M showing median cuticular strip with scaly ornamentation and lateral setal fringes. (Q) Cuticular triangle with

lateral cuticular fringes and V-shaped median strip, seen in side-view. Slide numbers and England Finder coordinates listed in data S1. Scale bars: 50 µm except in C, F, H, J (5 µm).

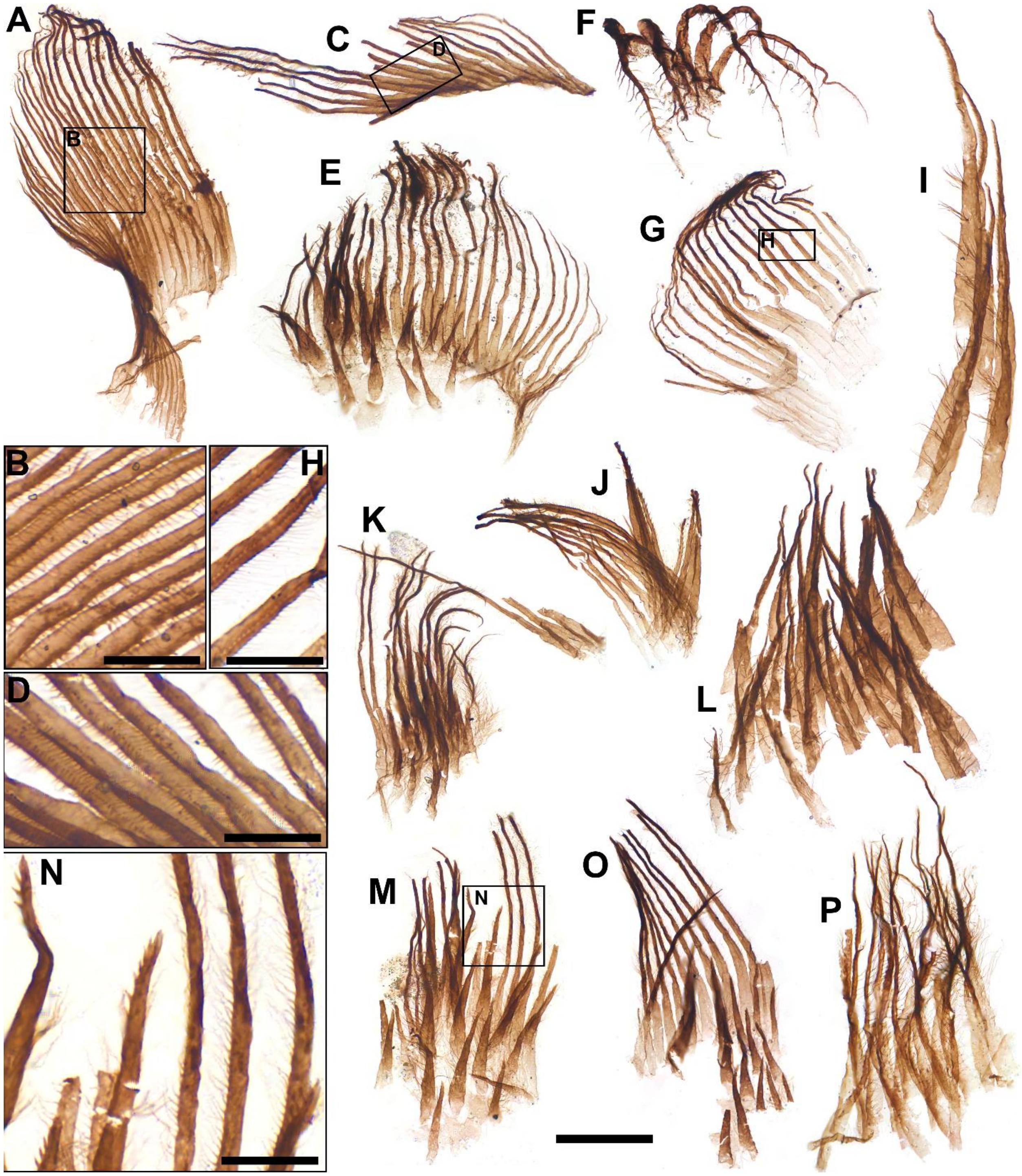

Fig. S10.

Additional setal arrays from the Bright Angel Biota. (A, C, E, G, O) Coplanar plumose arrays. (B, D, H) Details of boxed areas in A, C, and G showing biseriate lateral setules on closely spaced plumose setae. (F) Cluster of setae bearing widely spaced setule pairs. (I) Setae bearing widely spaced tufts of setules and distal comb-like edge of sense setules. (J) Partly preserved plumose array with flanking papposerrate setae. (K, P) Clusters of pappose setae. (L) Array of setae bearing widely spaced tufts of setules and distal comb-like edge of sense setules. (M) Cluster or plumose setae with flanking papposerrate setae, part of a probable filter plate. (N) Detail of boxed area in M showing lateral setules of plumose setae and terminations of papposerrate setae. Slide numbers and England Finder coordinates listed in data S1. Scale bars: 25 μm except in B, H, D, and N (5 μm).

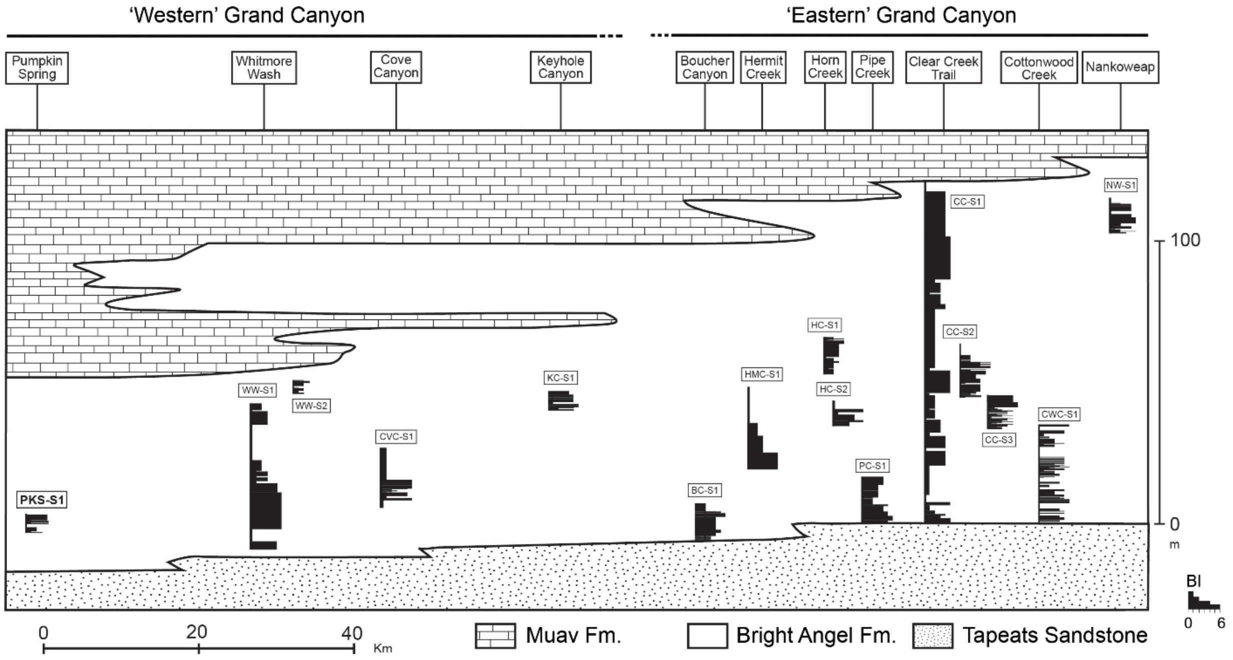

Fig. S11. Bioturbation levels across the Bright Angel Formation.
Schematic west to east cross-section of the Bright Angel Formation within the Grand Canyon, showing a general perspective of the lateral and vertical locations of each bioturbation index corresponding to sections measured by Miller (*39*), as indicated by legend to the bottom right. See Appendix 1 (Eastern Grand Canyon) and Appendix 2 (Western Grand Canyon) of (*39*) for detailed information on each measured section.

Movie S1. (separate file). Reconstructed pharynx of *Kraytdraco spectatus* gen. et sp. nov.
Blender animation showing an artistic reconstruction of the eversion and inversion of the pharyngeal apparatus in *Kraytdraco spectatus* gen. et sp. nov. Artist Credit: Rhydian Evans.

Data S1. (separate file). Specimens catalogue.
Catalogue of studied SCFs with taxonomic attribution, sample provenance and accession numbers.

REFERENCES AND NOTES

1. D. H. Erwin, M. Laflamme, S. M. Tweedt, E. A. Sperling, D. Pisani, K. J. Peterson, The cambrian conundrum: Early divergence and later ecological success in the early history of animals. *Science* **334**, 1091–1097 (2011).

2. N. J. Butterfield, Secular distribution of Burgess-Shale-type preservation. *Lethaia* **28**, 1–13 (1995).

3. N. J. Butterfield, T. H. P. Harvey, Small carbonaceous fossils (SCFs): A new measure of early Paleozoic paleobiology. *Geology* **40**, 71–74 (2012).

4. R. R. Gaines, M. L. Droser, The paleoredox setting of Burgess Shale-type deposits. *Palaeogeogr. Palaeoclimatol. Palaeoecol.* **297**, 649–661 (2010).

5. R. R. Gaines, Burgess Shale-type preservation and its distribution in space and time. *Paleontol. Soc. Papers* **20**, 123–146 (2014).

6. M. L. Droser, S. Finnegan, The Ordovician Radiation: A follow-up to the Cambrian explosion? *Integr. Comp. Biol.* **43**, 178–184 (2003).

7. A. Izquierdo-López, J. B. Caron, The Cambrian Odaraia alata and the colonization of nektonic suspension-feeding niches by early mandibulates. *Proc. Biol. Sci.* **291**, 20240622 (2024).

8. J. B. Caron, C. Aria, Cambrian suspension-feeding lobopodians and the early radiation of panarthropods. *BMC Evol. Biol.* **17**, 29 (2017).

9. J. Vinther, M. Stein, N. R. Longrich, D. A. T. Harper, A suspension-feeding anomalocarid from the Early Cambrian. *Nature* **507**, 496–499 (2014).

10. R. R. Gaines, D. C. García-Bellido, J. B. Jago, P. M. Myrow, J. R. Paterson, The Emu Bay Shale: A unique early Cambrian Lagerstätte from a tectonically active basin. *Sci. Adv.* **10**, eadp2650 (2024).

11. F. Saleh, C. S. Qi, L. A. Buatois, M. G. Mángano, M. Paz, R. Vaucher, Q. F. Zheng, X. G. Hou, S. E. Gabbott, X. Y. Ma, The Chengjiang Biota inhabited a deltaic environment. *Nat. Commun.* **13**, 1569 (2022).

12. J. R. Ineson, J. S. Peel, Geological and depositional setting of the Sirius Passet Lagerstätte (Early Cambrian), North Greenland. *Can. J. Earth Sci.* **48**, 1259–1281 (2011).

13. D. A. T. Harper, E. U. Hammarlund, T. P. Topper, A. T. Nielsen, J. A. Rasmussen, T. Y. S. Park, M. P. Smith, The Sirius Passet Lagerstätte of North Greenland: A remote window on the Cambrian explosion. *J. Geol. Soc.* **176**, 1023–1037 (2019).

14. J. R. Foster, R. R. Gaines, Taphonomy and paleoecology of the 'middle' Cambrian (Series 3) formations in Utah's West Desert: Recent finds and new data. *UGA* **45**, 291–336 (2016).

15. J. P. Lin, Review of the depositional environment of the Kaili Formation (Cambrian Series 2-3 boundary interval: China). *Mem. Assoc. Australas. Palaeontol.* **37**, 131–149 (2009).

16. J. P. Lin, Y. L. Zhao, I. A. Rahman, S. H. Xiao, Y. Wang, Bioturbation in Burgess Shale-type Lagerstätten - Case study of trace fossil-body fossil association from the Kaili Biota (Cambrian Series 3), Guizhou, China. *Palaeogeogr. Palaeoclimatol. Palaeoecol.* **292**, 245–256 (2010).

17. T. H. P. Harvey, J. Ortega-Hernández, J. P. Lin, Y. L. Zhao, N. J. Butterfield, Burgess Shale-type microfossils from the middle Cambrian Kaili Formation, Guizhou Province, China. *Acta Palaeontol. Pol.* **57**, 423–436 (2012).

18. J. Kimmig, L. C. Strotz, S. R. Kimmig, S. O. Egenhoff, B. S. Lieberman, The Spence Shale Lagerstätte: An important window into Cambrian biodiversity. *J. Geol. Soc.* **176**, 609–619 (2019).

19. D. E. Garson, R. R. Gaines, M. L. Droser, W. D. Liddell, A. Sappenfield, Dynamic palaeoredox and exceptional preservation in the Cambrian Spence Shale of Utah. *Lethaia* **45**, 164–177 (2012).

20. S. R. Hammersburg, S. T. Hasiotis, , R. A. Robison, Ichnotaxonomy of the Cambrian Spence Shale member of the Langston Formation, Wellsville Mountains, northern Utah, USA. *Paleont. Contr.* **2018**, 1–66. (2018).

21. A. F. Whitaker, J. D. Schiffbauer, D. E. G. Briggs, W. W. Leibach, J. Kimmig, Preservation and diagenesis of soft-bodied fossils and the occurrence of phosphate-associated rare earth elements in the Cambrian (Wuliuan) Spence Shale Lagerstätte. *Palaeogeogr. Palaeoclimatol. Palaeoecol.* **592**, 110909 (2022).

22. G. Mussini, Y. P. Veenma, N. J. Butterfield, A peritidal Burgess-Shale-type fauna from the middle Cambrian of western Canada. *Palaeontology* **68**, e70001 (2025).

23. T. H. P. Harvey, N. J. Butterfield, Great Canadian Lagerstätten 2. Macro- and Microfossils of the Mount Cap Formation (Early and Middle Cambrian, Northwest Territories). *Geosci. Can.* **38**, 165–173 (2011).

24. T. H. P. Harvey, M. I. Vélez, N. J. Butterfield, Exceptionally preserved crustaceans from western Canada reveal a cryptic Cambrian radiation. *Proc. Natl. Acad. Sci. U.S.A.* **109**, 1589–1594 (2012).

25. T. H. Harvey, M. I. Vélez, N. J.Butterfield*,* S. G. Survey, Ed. (Saskatchewan Geological Survey, 2012), vol. 1, pp. 1–8.

26. E. Wallet, B. Slater, S. Willman, J. S. Peel, Small Carbonaceous Fossils (SCFs) from North Greenland: New light on metazoan diversity in early Cambrian shelf environments. *Pap. Palaeontol.* **7**, 1403–1433 (2021).

27. P. A. Parsons, Competition versus abiotic factors in variably stressful environments: Evolutionary implications. *Oikos* **75**, 129–132 (1996).

28. G. J. Vermeij, On escalation. *Annu. Rev. Earth Planet. Sci.* **41**, 1–19 (2013).

29. K. L. Voje, O. H. Nolen, L. H. Liow, N. C. Stenseth, The role of biotic forces in driving macroevolution: Beyond the Red Queen. *Proc. R. Soc. B* **282**, 20150186 (2015).

30. G. J. Vermeij, The evolutionary interaction among species: Selection, escalation, and coevolution. *Annu. Rev. Ecol. Syst.* **25**, 219–236 (1994).

31. G. J. Vermeij, *Evolution and Escalation: An Ecological History of Life* (Princeton University Press, 1987).

32. R. Wood, A. Y. Zhuravlev, Escalation and ecological selectively of mineralogy in the Cambrian Radiation of skeletons. *Earth-Sci. Rev.* **115**, 249–261 (2012).

33. D. H. Erwin, Macroevolution of ecosystem engineering, niche construction and diversity. *Trends Ecol. Evol.* **23**, 304–310 (2008).

34. R. D. Bicknell, N. E. Campione, G. A. Brock, J. R. Paterson, Adaptive responses in Cambrian predator and prey highlight the arms race during the rise of animals. *Curr. Biol.* **35**, 882–888.e2 (2025).

35. F. A. Sundberg, Trilobite biostratigraphy of the Cambrian 5 and Drumian stages, series 3 (Laurentian Delamaran, Topazan, and Marjuman stages, Lincolnian Series) of the lower Emigrant Formation at Clayton Ridge, Esmeralda County, Nevada. *J. Paleontol.* **92**, 1–44 (2018).

36. C. Dehler, F. Sundberg, K. Karlstrom, L. Crossey, M. Schmitz, S. Rowland, J. Hagadorn, The cambrian of the Grand Canyon: Refinement of a classic stratigraphic model. *GSA Today* **34**, 4–11 (2024).

37. C. T. Baldwin, P. K. Strother, J. H. Beck, E. Rose, Palaeoecology of the Bright Angel Shale in the eastern Grand Canyon, Arizona, USA, incorporating sedimentological, ichnological and palynological data. *Geol. Soc. Spec. Publ.* **228**, 213–236 (2004).

38. E. C. Rose, Depositional Environments and History of the Cambrian Tonto Group, Grand Canyon, Arizona, thesis, Northern Arizona University, Flagstaff, Arizona (2003).

39. A. E. Miller, Ichnology of the Bright Angel Shale Formation, Grand Canyon, Arizona: Indicators for Middle Cambrian Paleoecology, thesis, Northern Arizona University, Flagstaff, Arizona (2019).

40. K. E. Karlstrom, M. T. Mohr, M. D. Schmitz, F. A. Sundberg, S. M. Rowland, R. Blakey, J. R. Foster, L. J. Crossey, C. M. Dehler, J. W. Hagadorn, Redefining the Tonto Group of Grand Canyon and recalibrating the Cambrian time scale. *Geology* **48**, 425–430 (2020).

41. K. Karlstrom, J. Hagadorn, G. Gehrels, W. Matthews, M. Schmitz, L. Madronich, J. Mulder, M. Pecha, D. Giesler, L. Crossey, Cambrian Sauk transgression in the Grand Canyon region redefined by detrital zircons. *Nat. Geosci.* **11**, 438–443 (2018).

42. E. D. McKee, C. E. Resser, *Cambrian history of the Grand Canyon region* (Carnegie Institution of Washington, 1945), vol. **563**, pp. 3–168.

43. H. R. Wanless, "Carbonate tidal flats of the Grand Canyon Cambrian" in *Tidal Deposits*, R. N. Ginsburg, Ed. (Springer, 1975), pp. 269–277.

44. A. E. Miller, L. Marchetti, H. Francischini, S. G. Lucas, "Paleozoic invertebrate ichnology of Grand Canyon National Park" in *Grand Canyon National Park Centennial Paleontological Resource Inventory,* V. L. Santucci, J. S. Tweet, Eds. (Utah Geological Association, ed. 1, 2020), chap. 8, pp. 131–170.

45. J. A. MacEachern, S. G. Pemberton, M. K. Gingras, K. L. Bann, "Ichnology and facies models" in *Facies Models*, N. P. James, R. W. Dalrymple, Ed. (Geological Association of Canada, 2010), pp. 19–58.

46. R. J. Foster, "Trilobites and other fauna from two quarries in the Bright Angel Shale (Middle Cambrian, Series 3; Delamaran), Grand Canyon National Park, Arizona." in *Cambrian Stratigraphy and Paleontology of Northern Arizona and Southern Nevada,* J. S. Hollingsworth, Sundberg, F.A. & Foster, J.R., Ed. (Museum of Northern Arizona Bulletin, 2011), vol. 67, pp. 99–120.

47. L. S. Lassiter, J. S. Tweet, F. A. Sundberg, J. R. Foster, P. J. Bergman, Paleozoic invertebrate paleontology of Grand Canyon National Park. *UGA* **1**, 73–103 (2021).

48. B. Slater, M. S. Bohlin, Animal origins: The record from organic microfossils. *Earth-Sci Rev* **232**, 104107 (2022).

49. A. Schmidt-Rhaesa, "Priapulida." in *Handbook of Zoology: Gastrotricha, Cycloneuralia and Gnathifera. Vol. 1: Nematomorpha, Priapulida, Kinorhynca, Loricifera.,* Schmidt-Rhaesa, Ed. (De Gruyter, 2012).

50. J. V. Wernström, B. J. Slater, M. V. Sorensen, D. Crampton, A. Altenburger, Geometric morphometrics of macro- and meiofaunal priapulid pharyngeal teeth provides a proxy for studying Cambrian "tooth taxa". *Zoomorphology* **142**, 411–421 (2023).

51. M. R. Smith, T. H. P. Harvey, N. J. Butterfield, The macro- and microfossil record of the Cambrian priapulid *Ottoia*. *Palaeontology* **58**, 705–721 (2015).

52. S. Conway Morris, Fossil priapulid worms. *Spec. Pap. Palaeontol.* **20**, 1–95 (1977).

53. V. Storch, Higgins, R. P., Malakhov, V. V., Adrianov, A. V., Microscopic anatomy and ultrastructure of the introvert of *Priapulus caudatus* and *P. tuberculatospinosus* (Priapulida). *J. Morphol.* **220**, 281–293 (1994).

54. D. Wang, J. Vannier, X. G. Yang, J. Sun, Y. F. Sun, W. J. Hao, Q. Q. Tang, P. Liu, J. Han, Cuticular reticulation replicates the pattern of epidermal cells in lowermost Cambrian scalidophoran worms. *Proc. Biol. Sci.* **287**, 20200470 (2020).

55. G. D. Edgecombe, S. Richter, G. D. Wilson , The mandibular gnathal edges: Homologous structures throughout Mandibulata? *Afr. Invertebr.* **44**, 115–135 (2003).

56. G. Mura, Scanning electron microscopic study of the molar surfaces of the mandibles of *Chirocephalus diaphanus* Prévost (Anostraca). *Crustaceana* **60**, 178–185 (1991).

57. G. Mura, Pattern of mandibular morphology in Anostraca with some taxonomical remarks. *Crustaceana* **69**, 129–154 (1996).

58. T. H. P. Harvey, N. J. Butterfield, A new species of early Cambrian arthropod reconstructed from exceptionally preserved mandibles and associated small carbonaceous fossils (SCFs). *Pap. Palaeontol.* **8**, e1458 (2022).

59. T. H. P. Harvey, N. J. Butterfield, Sophisticated particle-feeding in a large Early Cambrian crustacean. *Nature* **452**, 868–871 (2008).

60. N. J. Butterfield, An Early Cambrian radula. *J. Paleontol.* **82**, 543–554 (2008).

61. A. H. Scheltema, The original molluscan radula and progenesis in Aplacophora revisited. *J. Nat. Hist.* **48**, 2855–2869 (2014).

62. B. J. Slater, T. H. P. Harvey, R. Guilbaud, N. J. Butterfield, A cryptic record of Burgess Shale-type diversity from the Early Cambrian of Baltica. *Palaeontology* **60**, 117–140 (2017).

63. G. Mussini, N. J. Butterfield, Exotic cuticular specialisations in a Cambrian scalidophoran. *Proc. R. Soc. B* **292**, 20242806 (2025).

64. M. V. Sørensen, H. S. Rho, W. G. Min, D. Kim, A new recording of the rare priapulid *Meiopriapulus fijiensis*, with comparative notes on juvenile and adult morphology. *Zool. Anz.* **251**, 364–371 (2012).

65. E. Kirsteuer, & Ruetzler, K., Additional notes on *Tubiluchus corallicola* (Priapulida), based on scanning electron microscope observations. *Mar. Biol.* **20**, 78–87 (1973).

66. C. B. Calloway, Morphology of the introvert and associated structures of the priapulid *Tubiluchus corallicola* from Bermuda. *Mar. Biol.* **31**, 161–174 (1975).

67. V. Storch, R. P. Higgins, M. P. Morse, Internal anatomy of *Meiopriapulus fijiensis* (Priapulida). *Trans. Am. Microsc. Soc.* **108**, 245–261 (1989).

68. A. Schmidt-Rhaesa, S. Panpeng, H. Yamasaki, Two new species of *Tubiluchus* (Priapulida) from Japan. *Zool. Anz.* **267**, 155–167 (2017).

69. V. Storch, G. Alberti, R. M. Rosito, F. B. Sotto, Some ultrastructural observations on *Tubiluchus philippinensis* (Priapulida), A new faunal element of Philippine coastal caters. *Philipp. Sci.* **22**, 144–156 (1985).

70. J. Raeker, K. Worsaae, A. Schmidt-Rhaesa, New morphological structures of Priapulus caudatus, Lamarck 1816 (Priapulida) and analysis of homologous characters across macroscopic priapulids. *Zool. Anz.* **312**, 135–152 (2024).

71. J. Yang, M. R. Smith, X. G. Zhang, X. Y. Yang, Introvert and pharynx of *Mafangscolex*, a Cambrian palaeoscolecid. *Geol. Mag.* **157**, 2044–2050 (2020).

72. X. Shi, R. J. Howard, G. D. Edgecombe, X. Hou, X. Y. Ma, *Tabelliscolex* (Cricocosmiidae: Palaeoscolecidomorpha) from the early Cambrian Chengjiang Biota and the evolution of seriation in Ecdysozoa. *J. Geol. Soc.* **179**, doi.org/10.1144/jgs2021-060 (2022).

73. G. Mura, Morphological features of the mandible related to feeding habits of some Anostraca species. *Crustaceana* **68**, 83–102 (1995).

74. S. Richter, A comparison of the mandibular gnathal edges in branchiopod crustaceans: Implications for the phylogenetic position of the Laevicaudata. *Zoomorphology* **123**, 31–44 (2004).

75. H. G. Cannon, On the feeding mechanism of the Branchiopoda. *Philos. Trans. R. Soc. B Biol. Sci.* **222**, 267–339 (1933).

76. J. Olesen, Phylogeny of Branchiopoda (Crustacea)-character evolution and contribution of uniquely preserved fossils. *Arthropod Syst. Phylogeny.* **67**, 3–39 (2009).

77. D. Walossek, "The Upper Cambrian Rehbachiella and the phylogeny of Branchiopoda and Crustacea" in *The Upper Cambrian Rehbachiella and the phylogeny of Branchiopoda and Crustacea* (Fossils and Strata, Scandinavian University Press, 1993), pp. 1–202.

78. G. Fryer, Studies on the functional morphology and biology of the Notostraca (Crustacea: Branchiopoda). *Philos. Trans. R. Soc. Lond. B Biol. Sci.* **321**, 27–124 (1988).

79. N. J. Butterfield, Burgess Shale-type fossils from a Lower Cambrian Shallow-Shelf sequence in Northwestern Canada. *Nature* **369**, 477–479 (1994).

80. W. M. Hamner, Biomechanics of filter feeding in the Antarctic krill *Euphausia superba*: Review of past work and new observations. *J. Crustac. Biol.* **8**, 149–163 (1988).

81. G. D. Edgecombe, A new genus of henicopid centipede (Chilopoda: Lithobiomorpha) from New Caledonia. *Mem. Queensl. Mus.* **49**, 269–284 (2003).

82. S. Yamada, Functional importance of the mandibular skeleto-muscular system bivalved arthropod *Heterocypris incongruens* (Crustacea, Ostracoda, Cyprididae). *Sci. Nat.* **109**, 37 (2022).

83. B. K. Sullivan, C. B. Miller, W. T. Peterson, A. H. Soeldner, A scanning electron microscope study of the mandibular morphology of boreal copepods. *Mar. Biol.* **30**, 175–182 (1975).

84. N. J. Butterfield, Palaeoenvironmental distribution of Proterozoic microfossils, with an example from the Agu Bay Formation, Baffin Island. *Palaeontology* **35**, 943–957 (1992).

85. N. J. Butterfield, Plankton ecology and the Proterozoic-Phanerozoic transition. *Paleobiology* **23**, 247–262 (1997).

86. G. Fryer, G. Boxshall, The feeding mechanisms of *Lynceus* (Crustacea: Branchiopoda: Laevicaudata), with special reference to *L. simiaefacies* Harding. *Zool. J. Linn. Soc.* **155**, 513–541 (2009).

87. M. R. Smith, Mouthparts of the Burgess Shale fossils *Wiwaxia* and *Odontogriphus*: Implications for the ancestral molluscan radula. *Proc Biol Sci.* **279**, 4287–4295 (2012).

88. A. H. Scheltema, K. Kerth, A. M. Kuzirian, Original molluscan radula: Comparisons among Aplacophora, Polyplacophora, Gastropoda, and the Cambrian fossil. *J. Morphol.* **257**, 219–245 (2003).

89. J. Vinther, E. A. Sperling, D. E. G. Briggs, K. J. Peterson, A molecular palaeobiological hypothesis for the origin of aplacophoran molluscs and their derivation from chiton-like ancestors. *Proc. Biol. Sci.* **279**, 1259–1268 (2012).

90. S. Conway Morris, The Middle Cambrian metazoan *Wiwaxia corrugata* (Matthew) from the Burgess Shale and Ogygopsis Shale, British Columbia, Canada. *Philos. Trans. R. Soc. Lond. B Biol. Sci.* **307**, 507–582 (1985).

91. L. Salvini-Plawen, "The Structure and Function of Molluscan Digestive Systems" in *The Mollusca,* vol. ***11****. Form and function,* M. Clarke, Trueman, E., Ed. (Academic, San Diego, 1988), pp. 301–379.

92. A. Seilacher, J. W. Hagadorn, Early molluscan evolution: Evidence from the trace fossil record. *Palaios* **25**, 565–575 (2010).

93. D. Knaust, The ichnogenus *Teichichnus* Seilacher, 1955. *Earth-Sci. Rev.* **177**, 386–403 (2018).

94. D. Díez-Canseco, L. A. Buatois, M. G. Mángano, J. Cuevas-González, M. I. Benito, Unlocking the architecture of the colonization window: Ichnofabrics from upper cretaceous tide-influenced meander-loop deposits. *Palaios* **38**, 173–187 (2023).

95. D. G. Keighley, R. K. Pickerill, Commentary: The ichnotaxa *Palaeophycus* and *Planolites*: Historical perspectives and recommendations. *Ichnos* **3**, 301–309. (1995).

96. A. Ichaso, L. A. Buatois, M. G. Mangano, P. Thomas, D. Marion, Assessing the expansion of the Cambrian Agronomic Revolution into fan-delta environments. *Sci. Rep.* **12**, 14431 (2022).

97. A. Ichaso, "Stratigraphy, Sedimentology, and Ichnology of the Middle Cambrian to Lower Ordovician Deposits in Subsurface Western Canada (Doctoral Dissertation)", thesis, University of Saskatchewan, Saskatoon, Canada (2024).

98. F. Y. Chen, G. A. Brock, Z. L. Zhang, B. Laing, X. Y. Ren, Z. F. Zhang, Brachiopod-dominated communities and depositional environment of the Guanshan Konservat-Lagerstätte, eastern Yunnan, China. *J. Geol. Soc.* **178**, doi.org/10.1144/jgs2020-04 (2021).

99. A. Ichaso, L. Buatois, M. G. Mángano, "Paleoenvironmental Interpretations of the Basal Sandstone Unit and the Earlie Formation in Alberta and Saskatchewan Subsurface: Integration of Sedimentological and Ichnological Datasets." in *2022 Reports And Maps* (2022), vol. 1.

100. L. Buatois, M. G. Mángano, Paleoenvironmental variability of the Lower Paleozoic Earlie and Deadwood formations in subsurface Saskatchewan: A preliminary assessment. *Misc.Rep.* **1**, 8 (2013).

101. D. S. Herbers, R. B. MacNaughton, E. R. Timmer, M. K. Gingras, Sedimentology and ichnology of an Early-Middle Cambrian storm-influenced barred shoreface succession, Colville Hills, Northwest Territories. *Bull. Can. Pet. Geol.* **64**, 538–554. (2016).

102. M. J. Sommers, M. K. Gingras, R. B. Mac Naughton, K. M. Fallas, C. A. Morgan, Subsurface analysis and correlation of Mount Clark and lower Mount Cap formations (Cambrian), Northern Interior Plains, Northwest Territories. *Bull. Can. Pet. Geol.* **68**, 1–29 (2020).

103. C. Aria, J. B. Caron, A middle Cambrian arthropod with chelicerae and proto-book gills. *Nature* **573**, 586–589 (2019).

104. C. Aria, The origin and early evolution of arthropods. *Biol. Rev.* **97**, 1786–1809 (2022).

105. C. Aria, J. B. Caron, Burgess Shale fossils illustrate the origin of the mandibulate body plan. *Nature* **545**, 89–92 (2017).

106. A. Garm, L. Watling, in *Functional Morphology and Diversity, The Natural History of the Crustacea*. L. Watling, M. Thiel, Eds. (Oxford Academic, 2013).

107. M. R. Smith, A. Dhungana, Discussion on '*Tabelliscolex* (Cricocosmiidae: Palaeoscolecidomorpha) from the early Cambrian Chengjiang Biota and the evolution of seriation in Ecdysozoa'. *J. Geol. Soc.* **179**, doi.org/10.1144/jgs2021-111 (2022).

108. S. Q. Dornbos, J. Y. Chen, Community palaeoecology of the early Cambrian Maotianshan Shale biota: Ecological dominance of priapulid worms. *Palaeogeogr. Palaeoclimatol. Palaeoecol.* **258**, 200–212 (2008).

109. J. Vannier, I. Calandra, C. Gaillard, A. Zylinska, Priapulid worms: Pioneer horizontal burrowers at the Precambrian-Cambrian boundary. *Geology* **38**, 711–714 (2010).

110. M. R. Smith, G. M. G. Hughes, M. C. Vargas, F. de La Parra, Sclerites and possible mouthparts of *Wiwaxia* from the temperate palaeolatitudes of Colombia, South America. *Lethaia* **49**, 393–397 (2016).

111. C. E. Richmond, D. L. Breitburg, K. A. Rose, The role of environmental generalist species in ecosystem function. *Ecol. Model.* **188**, 279–295 (2005).

112. D. A. Piechnik, S. P. Lawler, N. D. Martinez, Food-web assembly during a classic biogeographic study: Species' "trophic breadth" corresponds to colonization order. *Oikos* **117**, 665–674 (2008).

113. Z. H. Liu, T. J. Algeo, S. Arefifard, W. Wei, C. Brett, E. Landing, S. M. Lev, Testing the salinity of Cambrian to Silurian epicratonic seas. *J. Geol. Soc.* **181**, jgs2023-2217 (2024).

114. B. J. Slater, Cambrian 'sap-sucking' molluscan radulae among small carbonaceous fossils (SCFs). *Proc. R. Soc. B* **290**, 20230257 (2023).

115. G. Mussini, N. J. Butterfield, A microscopic Burgess Shale: Small carbonaceous fossils from a deeper water biota and the distribution of Cambrian non-mineralized faunas. *Proc. R. Soc. B* **292**, (2025).

116. T.-Y. S. Park, M. L. Nielsen, L. A. Parry, M. V. Sørensen, M. Lee, J.-H. Kihm, I. Ahn, C. Park, G. de Vivo, M. P. Smith, D. A. T. Harper, A. T. Nielsen, J. Vinther, A giant stem-group chaetognath. *Sci. Adv.* **10**, eadi6678 (2024).

117. D. Jablonski, J. J. Sepkoski, D. J. Bottjer, P. M. Sheehan, Onshore-Offshore Patterns in the Evolution of Phanerozoic Shelf Communities. *Science* **222**, 1123–1125 (1983).

118. S. Conway Morris, Burgess Shale-type faunas in the context of the ‘Cambrian explosion’: A review. *J. Geol. Soc. London* **149**, 631–636 (1992).

119. D. L. Martin, “Depositional systems and ichnology of the Bright Angel Shale (Cambrian), eastern Grand Canyon, Arizona”, thesis, Northern Arizona University, Flagstaff, AZ (1985).

120. A. A. Lane, S. J. Braddy, D. E. G. Briggs, D. K. Elliott, A new trace fossil from the Middle Cambrian of the Grand Canyon, Arizona, USA. *Palaeontology* **46**, 987–997 (2003).

121. M. R. Smith, N. J. Butterfield, A new view on *Nematothallus*: Coralline red algae from the Silurian of Gotland. *Palaeontology* **56**, 345–357 (2013).

122. B. Slater, Life in the Cambrian shallows: Exceptionally preserved arthropod and mollusk microfossils from the early Cambrian of Sweden. *Geology* **52**, 256–260 (2024).

123. J. S. Peel, C. B. Skovsted, E. Wallet, Morphology and ecology of the bradoriid arthropods and from the Cambrian (Series 2, Stage 4) of North Greenland (Laurentia). *Palz* **95**, 413–427 (2021).
